# Maximising the Impact of Speech and Language Therapy for Children With Speech Sound Disorder (The MISLToe‐SSD) Study: Developing a Core Outcome Set (COS) for Routine Data Collection From UK NHS Speech and Language Therapy Services

**DOI:** 10.1111/1460-6984.70188

**Published:** 2026-01-09

**Authors:** Helen Stringer, Sam Burr, Joanne Cleland, Sam Harding, Yvonne Wren, The MISLToe_SSD Expert Panel

**Affiliations:** ^1^ School of Education, Communication and Language Sciences Newcastle University Newcastle upon Tyne UK; ^2^ Bristol Speech and Language Therapy Research Unit, Bristol, UK; ^3^ Cardiff Metropolitan University Cardiff UK; ^4^ Department of Psychological Sciences and Health, University of Strathclyde Glasgow UK; ^5^ University of Bristol Bristol UK

**Keywords:** core outcome set (COS), Delphi, speech sound disorder (SSD), minimum dataset

## Abstract

**Background:**

Children with speech sound disorder (SSD) are at risk of long‐term adverse consequences if appropriate intervention is not provided in a timely way. Although there are interventions of proven efficacy, these are often not implemented with good fidelity in clinical practice. Children with SSD in the United Kingdom are commonly managed in care pathways within NHS and independent speech and language therapy services. It is not known which care pathways are most effective because there is currently no systematic recording or analysis of intervention outcomes for children with SSD.

**Aims:**

The objective of the MISLToe‐SSD study is to develop an evidence‐based protocol for collecting routine data on a large‐scale so that UK SSD care pathways can be evaluated for clinical‐ and cost‐effectiveness. The development of the core outcome set (COS) is reported here.

**Methods and Procedures:**

Following the Core Outcome Measures in Effectiveness Trials methodology, a modified Delphi process was used to reach a consensus on a COS for SSD interventions. The Delphi process comprised two online survey rounds and one online meeting. Anonymity between panel members was maintained during the online survey rounds. Round one required a consensus of ≥50%, rising to ≥75% in round two.

**Outcomes and Results:**

A group of 66 UK speech and language therapists identified as experts in SSD by their peers were recruited through specialist clinical and research networks. A long list of 30 outcome statements was reduced by consensus to a final list of seven outcomes with associated measurement instruments. Increased speech intelligibility was agreed as the primary outcome by 100% of panel members. Six secondary outcomes were identified.

**Conclusions and Implications:**

The final COS can be used in future research to evaluate care pathways and intervention effectiveness for children with SSD. Furthermore, it provides a basis for measuring outcomes in future intervention trials for SSD.

**WHAT THIS PAPER ADDS:**

*What is already known on this subject*

- Speech and language therapy services in the United Kingdom are ideally placed to contribute to large‐scale evaluations of services provided for children with speech sound disorder (SSD) due to the computerised routine collection of data related to client management and interventions. However, these routine data cannot currently be used to evaluate the effectiveness or efficiency of interventions for children with different subtypes of SSD due to a lack of uniformity in data collection. There are no agreed and validated outcomes, outcome measures, diagnostic protocols or agreed labels and definitions for the evidence‐based interventions that can be used by services across the United Kingdom.

*What this study adds to existing knowledge*

- Building on information from an umbrella review and practitioner workshops, a modified Delphi process with 66 SSD expert speech and language therapists from across the United Kingdom, was utilised to develop a core outcome set (COS) and a minimum dataset of common data elements.

*What are the potential or actual clinical implications of this study?*

- The COS and minimum dataset can be used by speech and language therapy services to collect routine data in a way that can contribute to large‐scale evaluations of the effectiveness and efficiency of interventions for children with SSD.

## 1 Introduction

### 1.1 Background and Objectives

In the United Kingdom, National Health Service (NHS) speech and language therapy is provided to children free at the point of access. Some speech and language therapy is also provided through independent providers at a cost to the user. It is estimated (Broomfield and Dodd 2004) that every year in the United Kingdom over 48,000 children with speech sound disorders (SSDs) receive speech and language therapy. However, since the COVID‐19 pandemic the number of children requiring speech and language therapy has risen sharply (Speech and Language UK (ICAN) 2021). This has been accompanied by a reduction in speech and language therapy services with vacancy levels across the United Kingdom in all children's services (both NHS and independent) as high as 47% (RCSLT and ASLTIP 2023). NHS England reported in January 2024 that the number of children and young people on speech and language therapy waiting lists was 73,635 (Statistics Community Health Services Waiting Listsn.d.), with over 3000 waiting over 18 weeks. It is estimated that approximately 30% of these will have SSD (Broomfield and Dodd 2004), equating to approximately 22,000 children with SSD in England alone waiting for speech and language therapy assessment and intervention.

The most significant negative impact of SSD is on a child's intelligibility, making it difficult for others to understand what the child is saying and consequently reducing communicative participation. However, there are also speech and language processing consequences that impact on word learning and morphology (Stackhouse and Wells 1997), reducing language capability. Children with SSD that is not resolved before they start literacy instruction, usually around five years of age in the United Kingdom, are at a disadvantage compared to their typically developing peers. They are at risk of reading and spelling difficulties with long‐term consequences for access to the curriculum (Bird et al. 1995; Wren et al. 2021), of poor social outcomes and poor mental health (Wren et al. 2023).

Speech and language therapy for children is not delivered in a uniform way across the United Kingdom. Self‐report by speech and language therapy services reveals that some offer care pathways for children with different types of speech, language and communication disorder for example, SSD pathway, preschool language pathway; some present a local offer that includes all children for example, assessment and a specified number of intervention sessions; others work closely with local educational settings to pool resources and provide a combined public health and specialist service; some enter in to service level agreements with educational settings to provide bought‐in services over and above the local offer; most work closely with parents/caregivers; most have assistant SLTs who carry out supervised delegated interventions. This is not an exhaustive list. For ease of reading, any type of service provision for children with SSD will be referred to as an SSD care pathway in this paper.

The need to identify which SSD care pathways are associated with the best outcomes and are most cost‐effective is more pressing than ever. The evidence base for SSD interventions indicates that there are a number of efficacious interventions available to speech and language therapists (SLTs) (Baker and McLeod 2011; Wren et al. 2018). However, these may not all translate into effective interventions when delivered within speech and language therapy service resource constraints. For example, some services are required to deliver a set number of assessment and/or intervention sessions; others try to manage large numbers of referrals with inadequate resources providing reduced and ineffective dosages (Hegarty et al. 2018). Furthermore, despite the availability of, for example, therapy outcome measures (TOMs) (Enderby and John 2019a, 2019b), outcome data are not routinely collected by speech and language therapy services (Roulstone et al. 2012); therefore, it is not known which intervention or combination of interventions is most effective in routine clinical practice.

The objective of the Maximising the Impact of Speech and Language Therapy for children with Speech Sound Disorder (MISLToe_SSD) study is to develop and trial a robust methodology to enable collection of routine intervention and outcome information from speech and language therapy services in the United Kingdom. This will enable the establishment of a large‐scale dataset that can be mined to discover the most effective interventions and delivery methods for children and young people with different types of SSD. To do this in a cost‐ and time‐efficient way, a core outcome set (COS) for SSD with an agreed minimum dataset of known confounders and covariates to aid accurate analysis of the COS data are required. Although the TOMs can be used to demonstrate progress over time (Hesketh et al. 2023) it would not meet the aims of the MISLToe‐SSD study as an outcome set or measure. The TOMs provide a subjective before and after score for comparison but do not collect quantitative data such as number of error patterns, percentage consonant correct (PCC), or details of the intervention such as type and dosage, all essential elements of the large dataset that is the ultimate aim of MISLToe‐SSD. The need for a bespoke COS to meet the requiremnts for specific purposes and populations is reflected in recent COS development for a group of schools in Ontario, Canada (Cahill et al. 2024). None of the existing COS relate specifically to SSD or facilitate data collection to populate a large‐scale dataset that could be used to evaluate the effectiveness of different interventions for children with SSD.

This paper reports one strand of the MISLToe‐SSD phase one study. A modified Delphi process was planned to reach consensus in two areas: (1). A COS and outcome measures for interventions for SSD that will facilitate data collection from speech and language therapy services across the United Kingdom; (2). A minimum dataset of information collected to support optimal use of the COS in research. Three other strands of MISLToe‐SSD phase one were also conducted to inform the Delphi process. First, an umbrella review of existing outcome tools for SSD was carried out (Harding et al. 2023, 2024), extracting outcomes, outcome measures and interventions from studies inlcuded in 16 studies and two reports. These data were used to inform the following stages of the study. The review established a distinction between the outcome concept (where change may be observed) and the outcome tool (the means to measure that change). Second, two participatory workshops to determine a diagnostic protocol and defined list of interventions to be used with the resulting COS were conducted with representatives from NHS speech and language therapy services (Cleland et al. 2025).

A third strand of Patient Public Involvement and Engagement (PPIE) ran in parallel to the previous two strands. Individuals with lived experience of SSD as parents or children were involved through interviews and emotional mapping activities respectively (Harding et al.,in review). Young People's Advisory Groups (YPAGs) were consulted on three occasions. YPAGs are trained groups of young people who inform health services research, supported by the UK National Institute for Health Research (NIHR). They include young people with and without experience of SSD and other types of language and communication disorders. Information about important outcomes, acceptability of timing and types of intervention and data collection was collected and informed the participatory workshops and Delphi process.

The Delphi technique is an iterative process in which a group of panellists are individually asked a set series of questions and provided with controlled feedback. The feedback allows panellists to reappraise their views with knowledge of the responses of the whole group (Thangaratinam and Redman 2005; Trevelyan and Robinson 2015). It is a useful technique in areas where expert consensus based on the panellists’ experience and knowledge is required. It allows for a decision‐making process in which all contributors are allowed an equal voice and the group consensus, rather than any individual's opinion, has most influence on the outcome. This study employed a modified Delphi process, with the usual first Delphi round of open‐ended outcome statement generation replaced with outcomes from previous activity in MISLToe‐SSD (Cleland et al. 2025; Harding et al. 2023, 2024) incorporated into a series of statements. The aim of this COS and minimum dataset is to facilitate routine data collection from UK NHS speech and language therapy services to be used in future research into the effectiveness of SSD care pathways. Phase two of MISLToe‐SSD will test the feasibility of routine use of the COS and minimum dataset and make adjustments to the COS or minimum dataset as required for planned large‐scale utilisation in MISLToe‐SSD phase three. In addition, the data collected will allow speech and language therapy services to evaluate their own SSD care pathway and make any desired adjustments to increase clinical and cost‐effectiveness. Each core outcome will comprise a defined SSD outcome (e.g., speech intelligible to strangers; increased percentages of consonants correct), an agreed measurement instrument (assessment) and an agreed statement of measurement/assessment timing during the child's progress through an SSD pathway of care (Trevelyan and Robinson 2015). The minimum dataset will ensure consistent collection of common data elements that will provide for example, accurate and consistent demographic data for panel members, that will support interpretation of COS data.

To ensure clarity of reporting, the COS‐Star reporting framework (Williamson et al. 2017) (see supplementary material 1) was followed for this paper.

## 2 Methods

The study was registered on the core outcome measures in effectiveness trials (COMET) Database (https://www.comet‐initiative.org/Studies, accessed 01/06/2024) prior to commencement. The original protocol for the workstream reported here is in supplementary material 6.

### 2.1 Participants

The expert panel comprised UK‐based SLTs who were clinicians and/or researchers in SSD. This aligned with the purpose of the MISLToe‐SSD study to formulate a COS for use by UK speech and language therapy services in routine data collection for evaluation of the effectiveness of SSD care pathways. Panel members were recruited by purposive sampling through specialist SLT, academic, and research networks.

Service delivery through the NHS compared to insurance or private models in other countries led to the focus on the United Kingdom. However, within speech and language therapy and SSD research, there is extensive international collaboration and some willingness to reach consensus on terminology and intervention approaches (Murphy 2019; Roulstone et al. 2012). We also recruited a small international expert panel to comment on the statements put forward in the modified Delphi process (reported separately). This will in turn inform future international research collaborations using the MISLToe‐SSD methodology to establish COS in languages other than English and countries other than the United Kingdom. Invitation emails with a link to an online participant information sheet (PIS) and consent form (JISC online surveys) were sent to known eligible SLTs and for cascading to the Association of SLTs in Independent Practice (ASLTiP); to the chairpersons of the United Kingdom and Ireland Childhood Speech Sound Disorder Research Network (CSDRN), the SSD Clinical Excellence Networks (CENs) and the Committee of Higher Education Institution Speech and Language Therapy Degree Programme Leads (CREST).

The knowledge and experience required of the panel members in relation to SSD was made explicit during the recruitment and consent process. At the start of the online Participant Information Sheet (PIS) and consent survey there were two screening questions which served to remove anyone who was not an SLT or who was not an expert or specialist in SSD. Anyone not meeting these criteria was immediately routed to the final page, thanking them for their interest. SLTs who indicated that others in their workplace or the wider profession considered them to be an expert or specialist in SSD were directed to the PIS and consent form. Information was provided explaining the project and Delphi process, what would be required of them, the timing and time commitment. Informed consent was obtained from all panel members.

There is no recommended ideal number of panel members for a Delphi study (Hasson et al. 2000; Thangaratinam and Redman 2005). We therefore set a pragmatic recruitment target of 60–70 panel members, allowing for attrition and ensuring a wide range of experience and knowledge whilst obtaining a volume of data manageable within study resources.

### 2.2 Procedure

#### 2.2.1 Statement Development

The outcomes of the umbrella review (Harding et al. 2024), participatory workshops (Cleland et al. 2025) and PPIE consultations were used as information sources to identify an initial list of outcomes, outcome measures/assessments and intervention types. The list of routinely collected common data elements to comprise a potential minimum dataset was developed by the research team, drawing on our experience as clinicians in the NHS and knowledge of existing online record keeping software used by speech and language therapy services in the United Kingdom.

#### 2.2.2 Consensus Process

**FIGURE 1:**
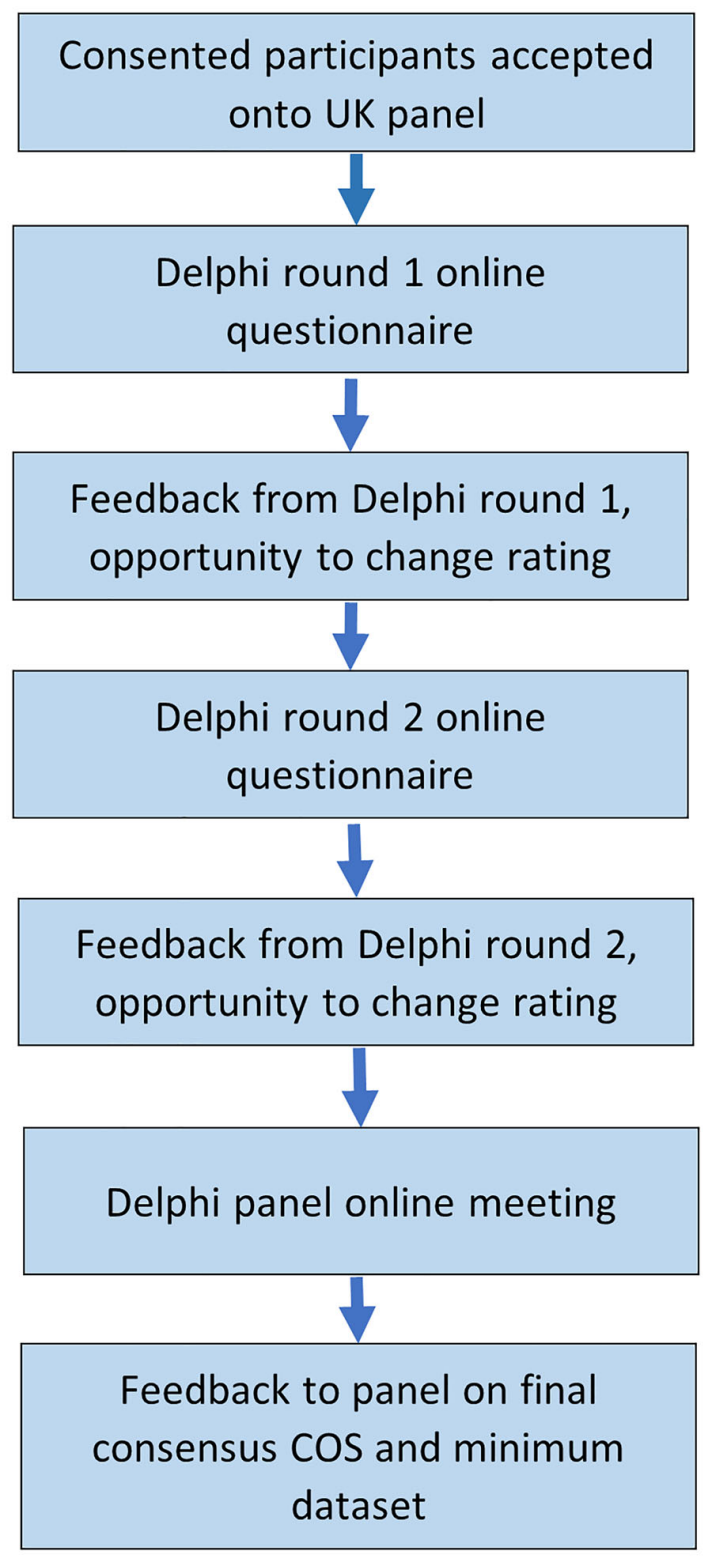
Flowchart for process of modified Delphi.

Following COMET methodology (Prinsen et al. 2014, 2016; Williamson et al. 2017) the modified online Delphi process was implemented using JISC online survey software. See Figure 1 for an overview of the modified Delphi process. There were two quasi‐anonymous Delphi rounds. In each round the panel members were anonymous to each other. All correspondence in rounds one and two was by email. Reminders were sent automatically after five and ten days to participants who had not yet responded. At this stage we aimed for a 90% response rate (Prinsen et al. 2016).

In an attempt to ensure all panel members were approaching the statements from the same or similar perspective, information was given about the population to which we would be applying the outcome, and the timescale over which the outcomes would be measured.

#### 2.2.3 Delphi Steering Group

The steering group for MISLToe‐SSD phase one comprised four academic SLTs, a Health Psychologist, an SLT clinical researcher, the CEO of a UK charity representing parents of children with SSD, an NHS speech and language therapy service manager and an NHS SLT specialising in developmental disorders. The MISLToe‐SSD core research team formed the steering group for the Delphi process. The whole study steering group provided input at various stages of the study, with suggestions and advice taken forward by the Delphi steering group.

#### 2.2.4 Modified Delphi Procedure

##### 2.2.4.1 Round One

Round one comprised potential outcomes, assessments and the minimum dataset (see supplementary material 2). There were 30 intervention outcome statements rated by panel members and considered as primary or secondary outcomes for children/families or SLTs (required response). Panel members were asked for any important outcomes not listed (optional response). A Likert scale was used for panel members to rate the importance of each outcome as a potential member of the COS. The Likert scale comprised the following five points:

1. Not at all important
2. Slightly important
3. Moderately important
4. Very important
5. Essential

Thirty‐seven different assessments were obtained from the umbrella review. These were listed in the survey with an initial question asking if the panel member knew the assessment (required response) to ensure that further information was based on knowledge rather than being speculative. Question logic required that positive responses to this initial question led to further questions about the assessment's suitability as an outcome measure for SSD (required response).The criteria for suitability were that the assessment would be used by panel members if they (a) had access to it, (b) considered it suitable as a baseline measure, and/or (c) an outcome measure, and (d) considered it suitable for measuring progress. Panel members were also asked for any assessments used in practice that were not listed (optional response). Language delay and disorder frequently co‐occur with SSD. Panel members were therefore asked if they also routinely assess the child's language skills (required response) and if so, what assessment they used (optional response).

Acknowledging that there may be circumstances when there are harmful effects or negative outcomes of intervention, panel members were asked if we should consider these (required response). Examples were given such as “if an inadequate assessment leads to an incorrect diagnosis”; “if the intervention is provided with reduced dosage”; “if the intervention provided is known to be ineffective for the given diagnosis or if there are more effective interventions that were not provided”. Panel members were invited to list harmful effects that they thought could occur (optional response).

Eighteen common data elements were listed for possible inclusion in the minimum data set (see supplementary material 3). A yes/no response was required against each of the 18 items in response to: (a) Is this item important to make sense of outcome data? and (b) Do you routinely record this item? Items with a ≥50% ’Yes’ response to Q1 were taken forward to the panel meeting for agreement.

Following round one, feedback was provided to panel members. This comprised a report of their own responses to round one (in PDF format) and a summary report of all responses with a representative sample of the free text responses. Panel members were asked to consider their initial rating in view of the group consensus and provide a revised rating if they wished to.

##### 2.2.4.2 Round Two

The second set of outcome statements comprised a reduced set of statements formed from items in round one rated *moderately important* or higher by ≥50% of panel members. These were presented in round two following the same procedure as round one (see supplementary material 4).

Assessments from round one that reached a threshold of ≥50% on all criteria went forward to round two. In addition, assessments mentioned by six or more panel members as used in practice were included in round two.

Individual reports and a group summary were provided to panel members after round two in the same format as for round one to allow panel members to modify their responses. After round two feedback was returned, those statements rated *moderately important* or higher or were identified in all assessment criteria by ≥75% of participants were taken forward to the panel meeting.

##### 2.2.4.3 Panel Meeting

The final COS and minimum dataset were agreed by a 3‐hour virtual (Zoom) meeting of the UK panel using the outcomes of round two. It was anticipated that at least 40 members of the panel would attend the meeting. A Padlet (https://padlet.com/) was made available for those who could not attend the panel but wished to continue to comment on the items. A report was sent to all panel members in advance of the meeting, setting out the consensus reached in the Delphi rounds and five points for discussion during the meeting or for comment on the Padlet. There was opportunity for whole‐panel discussion and dialogue in breakout groups that fed back to the whole panel.

The final set of statements regarding the outcomes were presented to the panel. Specifically, they were asked to discuss and come to agreement on the following: “The outcomes that reached ≥75 we propose to take forward into the COS. Some will only be applicable to some of the SSD caseload, whilst others can be universally applied. Two areas of outcome may prove to be more difficult to measure than the rest. We seek your opinion on how to label and measure these. They are:

- Items which relate to quality of life (QoL)
- Items which all relate to generalisation.” (extract from information provided to panel)

Panel members were able to put forward and listen to the arguments in support or otherwise of the statement. Outcomes that obtained a final consensus of ≥75% from the meeting and Padlet for inclusion in the COS were taken forward to be considered alongside available assessments.

Assessments taken forward from round two were discussed in the context of the outcomes. The necessity of being able to reliably measure all outcomes was discussed, and an agreement was reached on the final set of outcomes and outcome measures. Specifically, the panel were asked to reach a consensus agreement on assessments identified through rounds one and two.

The panel were asked about the inclusion of a language measure alongside assessments for SSD as they frequently co‐occur. Specifically, they were asked to discuss: *Should a language screen be a routine part of assessment of children presenting with SSD? If so, what is a suitable tool? Should we also use it to collect a connected speech sample, for example, a sentence repetition task?*

Common data elements that will form the minimum data set were presented to the panel in the context of the types of data software used in employing organisations. Issues relating to how diagnosis is recorded were discussed and the final minimum dataset was agreed upon.

##### 2.2.4.4 Analysing and Interpreting Results

Validity in the Delphi process can be difficult to establish (Thangaratinam and Redman 2005; Trevelyan and Robinson 2015). By maintaining anonymity in the first two rounds, we endeavoured to avoid bias caused by the influence of what could be perceived as more experienced or esteemed members. By giving panel members opportunity to modify their response in relation to others’ responses we are encouraging them to consider views and ideas that they may not have initially thought of, but that may align more closely with their experience. In this way, while remaining anonymous, they can all benefit from each other's experience and move towards consensus without one or two members having undue influence.

In round one, consensus was defined as statements rated *moderately important* or higher by ≥50% of panel members. In round two, consensus was defined as statements rated *moderately important* or higher by ≥75% of panel members (Keeney et al. 2006). The same thresholds were required for the assessments/measures and minimum dataset.

Quality assessment of outcome measures was conducted in alignment with the COnsensus‐based Standards for the selection of health status Measurement INstruments (COSMIN) protocol (https://www.cosmin.nl/tools/guideline‐selecting‐proms‐cos/ accessed 17/09/2022).

## 3 Results

### 3.1 Deviations From the Original Protocol

This paper reports workstream 3 of the MISLToe‐SSD phase one study. The original protocol for this workstream is in supplementary materials 6.

The following deviations from the original protocol occurred:

- The original intent was to recruit ≤60 participants in the Delphi expert panel. The interest in the project was greater than anticipated and a decision was made to keep all eligible panel members who consented to take part.
- The minimum dataset was included in round one, which is not explicit in the original protocol.

### 3.2 Participants/Panel Members

Seventy‐one SLTs consented to take part in the modified Delphi process. They all confirmed that they were considered by others in their workplace as expert or specialist in SSD. Of these, five did not respond to the first round of the Delphi and were therefore removed from future involvement. Sixty‐six panel members took part in Delphi round one. All four of the devolved UK countries (England, Scotland, Wales, Northern Ireland) were represented. Information about their employer(s); years qualified; years working with children with SSD and the type of work undertaken by the panel members in relation to children with SSD is set out in Table 1. Note that some had more than one employer and/or undertook more than one type of work.

**TABLE 1:** Employment experience and type of work with children with SSD for Delphi round one panel members (participants).

	Number of panel members (participants) in Delphi round one (*n* = 66) (%)
Employer for work with children with SSD	
NHS	57 (86%)
Higher education institution (university/college)	13 (20%)
Independent practice	8 (12%)
Local authority/government	2 (3%)
Other	1 (2%)

Years qualified as an SLT	
4–9 years	10 (15%)
10–19 years	26 (39%)
20–29 years	16 (24%)
Over 30 years	14 (21%)

Years working with children with SSD	
4–9 years	12 (18%)
10–19 years	29 (44%)
20–29 years	14 (21%)
Over 30 years	11 (17%)

Type of work with children with SSD	
Provide assessment and intervention for children with SSD	63 (95%)
Lead a clinical service for children with SSD	26 (39%)
Teach pre‐registration (undergraduate or postgraduate) students about SSD	15 (23%)
Conduct research in SSD assessment or intervention	13 (20%)

### 3.3 Delphi Round One

Sixty‐six panel members responded to the round one Delphi survey, with 16 taking the opportunity to amend their initial responses in the feedback survey.

#### 3.3.1 Rating the Importance of SSD Intervention Outcomes

The threshold for progression to the round two Delphi was 50% consensus at moderately important, very important or essential. The statements and percentage of responses towards the 50% threshold are in Table 3.

#### 3.3.2 Primary and Secondary Outcomes

Panel members were asked to make a judgement as to whether the outcomes might be a primary outcome for children and families and/or for speech and language therapists, see Table 3.

#### 3.3.3 Outcome Measures (Assessments)

Thirty‐seven assessments were presented for the expert panel, identified in the umbrella review (see Harding et al. 2024 for detail). Thirty‐four assessments were known by fewer than 49% of the expert panel members, with 22 of those assessments known by fewer than 10% of the panel.

The following assessments were known to over 50% of the expert panel:

1. Diagnostic Evaluation of Articulation and Phonology (DEAP, Dodd et al. 2002) (100%)
2. Edinburgh Articulation Test (EAT, Anthony et al. 1971) (50%)
3. Preschool and Primary Inventory of Phonological Awareness (PIPA, Dodd et al. 2000) (60%)

Of these, the DEAP would be used by 100% of panel members if they had access to it; 98% considered it suitable as a baseline and outcome measure; 95% considered it suitable for measuring progress. The EAT was not considered suitable as a measure and only 6% said they would use it if they had access to it. The PIPA was considered suitable as a baseline measure (52%) but only 49% would use it if available. Taking 50% as the threshold, the DEAP, including the DEAP Diagnostic Screen and the DEAP Toddler Phonology Test, therefore goes forward as the favoured assessment for baseline, outcome and progress assessment of articulation and phonology. The DEAP is the only assessment of phonology and articulation that has been standardised on a population representative of different areas of the United Kingdom. The diagnostic screen, which signposts more detailed assessment in three different areas of speech, makes the DEAP time‐efficient in data collection, establishing a differential diagnosis and signposting an appropriate intervention (see Waring and Knight 2013; Stringer et al. 2024, 11). In comparison to other available single word assessments of UK English speech, the DEAP is a reliable and straightforward way to collect PCC in this context (see Chapter 4, DEAP Manual, Dodd et al. 2002). For comparisons of PCC before and after intervention to be valid, the stimulus words should be of the same complexity (polysyllabic vs. monosyllabic; singleton vs. consonant clusters) and contain the same frequency of phonemes. Therefore the same word list is recommended. The DEAP standardisation supports clinical decision making regarding severity of SSD, by taking age as well as PCC into account.

A list of assessments that were not identified by the umbrella review, but were in use by panel members, was generated. This comprised 32 different assessments, some of which were suitable for specific subtypes of SSD for example, cleft palate ± lip, childhood apraxia of speech (CAS). The majority of assessments were only mentioned by one panel member. Those with six or more mentions, went forward to round two, see Table 2.

**TABLE 2:** Additional assessments.

Name of assessment	Number of mentions
CLEAR phonology screening assessment (Keeling and Keeling 2001)	29
NDP (Nuffield Dyspraxia Programme Assessment) (Williams and Stephens 2004)	28
STAP (South Tyneside Assessment of Phonology) (Armstrong and Ainley 1988)	23
NAPA (Newcastle Assessment of Phonological Awareness, including previous version APAD) (Stringer 2019)	14
ICS (Intelligibility in Context Scale) (McLeod et al. 2012b)	12
CAV‐ES (Clinical Assessment of Vowels‐ English ystems) (Bates et al. 2019)	6

#### 3.3.4 Language Assessment

The majority (92%) of panel members routinely assess some aspect of language when children are referred primarily for SSD. Sixty‐one percent routinely screen for language difficulties; 21% assess only if language delay or disorder is flagged; 11% assess language in depth. The assessments used by panel members were taken forward to inform protocol development for MISLToe‐SSD phase 2.

#### 3.3.5 Negative Consequences

A total 88% of panel members thought that negative outcomes should be included. The main harmful effects noted by panel members were inadequate assessment leading to incorrect diagnosis and intervention; giving inadequate dosage so that effectiveness was reduced or eliminated; adverse mental health impact on the child.

### 3.4 Minimum Dataset

A list of common data elements were presented to the panel members in round one. Panel members were asked if they considered the data elements important to interpret the outcome data. They were also asked if they routinely collected the data in their clinical practice. Details of their responses are in Supplementary Materials 3. The results were taken forward to panel meeting for more detailed discussion (see the following section).

### 3.5 Delphi Round Two

Sixty‐two of the original 66 panel members responded to the round two Delphi survey (6% attrition), with nine taking the opportunity to amend their initial responses in the feedback survey.

#### 3.5.1 Rating the Importance of SSD Intervention Outcomes

Seven statements were removed following round one, the remaining 23 statements went forward with no changes. Nineteen items met or exceeded the 75% threshold to go forward to the Delphi expert panel meeting, see Table 3.

**TABLE 3:** Rating of statements as moderately important, very important or essential, primary and secondary outcomes from Delphi rounds one and two.

SSD intervention outcomes	Round one. % at moderately important, very important or essential (50% threshold)	Round two. % at moderately important, very important or essential (75% threshold)	Children/ families primary outcome	SLT primary outcome	Secondary or other outcome
1. Increased speech intelligibility	100%	100%	64 (97%)	59 (89%)	0
2. Increased confidence when talking	91%	94%	58 (88%)	41 (62%)	8 (12%)
3. Improved QoL	89%	89%	52 (79%)	35 (53%)	13 (20%)
4. Improved communicative activity and participation	98%	97%	58 (88%)	47 (71%)	5 (8%)
5. Improved language	41%	n/a	6 (9%)	3 (5%)	58 (88%)
6. Improved vocabulary	35%	n/a	3 (5%)	3 (5%)	60 (91%)
7. Increased percentage consonants correct (PCC)	89%	94%	2 (3%)	49 (74%)	16 (24%)
8. Increase in percentage phonemes correct (PPC)	80%	85%	3 (5%)	47 (71%)	16 (24%)
9. Increase in percentage vowels correct (PVC)	86%	87%	3 (5%)	43 (65%)	22 (33%)
10. Increase in percentage of words correct (PWC)	73%	74%	8 (12%)	37 (56%)	27 (41%)
11. Increase in percentage of intelligible utterances (PIU)	79%	84%	15 (23%)	41 (62%)	21 (32%)
12. Decrease in proportion of errors (POE)	67%	65%	8 (12%)	38 (58%)	27 (41%)
13. Decrease in phonological variability	86%	84%	1 (2%)	43 (65%)	23 (35%)
14. Increased accuracy of target	88%	85%	26 (39%)	52 (79%)	11 (17%)
15. Increase in production of target sounds	89%	82%	29 (44%)	53 (80%)	11 (17%)
16. Increase in phonological awareness	86%	84%	6 (9%)	43 (65%)	21 (32%)
17. Increased stimulability	94%	89%	12 (18%)	51 (77%)	15 (23%)
18. Improved oromotor skills	24%	n/a	2 (3%)	8 (12%)	53 (80%)
19. Increase in number of phonemes	85%	89%	15 (23%)	45 (68%)	20 (30%)
20. Increase in egressive output	45%	n/a	2 (3%)	19 (29%)	47 (71%)
21. Generalisation across linguistic units	85%	84%	3 (5%)	27 (41%)	37 (56%)
22. Generalisation across word position	85%	87%	6 (9%)	32 (48%)	31 (47%)
23. Generalisation to a new context	82%	87%	18 (27%)	36 (55%)	27 (41%)
24. Generalisation of known sounds	73%	74%	15 (23%)	30 (45%)	34 (53%)
25. Generalisation of the intervention target	91%	89%	28 (42%)	49 (74%)	14 (21%)
26. Generalisation related to the target (e.g., generalisation to other phonemes within and across sound classes)	85%	85%	5 (8%)	35 (53%)	29 (44%)
27. Increased mean length of utterance (MLU)	26%	n/a	5 (8%)	1 (2%)	60 (91%)
28. Increase in percentage child utterance attempts that are fully intelligible from language sample	67%	71%	19 (29%)	31 (47%)	29 (44%)
29. Parent report on increased structural complexity	41%	n/a	8 (12%)	5 (8%)	54 (82%)
30. Parent report on increased phrase complexity	38%	n/a	9 (14%)	5 (8%)	54 (82%)

#### 3.5.2 Outcome Measures (Assessments)

This section of the Delphi process deviated from the original protocol as the survey did not match the format of the round one outcome measure survey. The DEAP (Dodd et al. 2002) was not further considered at this stage as it was unequivocally the measure of choice in round one. It is the only current speech sound assessment that is standardised on a UK population and signposts diagnostic label and intervention. Seven assessments were presented for consideration in Delphi round two. These included the six identified as additional assessments in previous rounds. The PIPA (Dodd et al. 2000), identified as a baseline measure in round one, was included to explore further the Panel's judgement on this assessment. The clear distinction between knowledge and use was blurred in this survey due to the removal of the round one question logic that only allowed panel members to make further responses if they were familiar with the assessment. The results here are more equivocal as panel members unfamiliar with the assessment could potentially comment on its usefulness.

A pragmatic approach to progression to the Expert Panel Meeting was therefore adopted. The DEAP (Dodd et al. 2002) comprises a diagnostic screen that signposts further assessment of articulation, phonology and inconsistency, with error pattern, percentage consonant, vowel and phoneme correct analysis. It is a more robust measure than the CLEAR Phonology Screening Assessment (Keeling and Keeling 2001) and STAP (Armstrong and Ainley 1988), neither of which are standardised or assess for inconsitencey and which were therefore dropped. The Clinical Assessment of Vowels‐ English Systems (CAV‐ES) (Bates et al. 2019) is a specialist assessment of vowel production. This was not taken forward as a core outcome measure but is recommended for children with unintelligible speech due to multiple vowel distortions. The ICS (McLeod et al. 2012a) is currently the only measure available that explicitly assesses intelligibility from the perspective of those who are in almost constant contact with the child (parent/caregiver). This was therefore taken forward. Of the two assessments of phonological awareness, the Newcastle Assessment of Phonological Awareness (NAPA) (Stringer 2019) received higher scores from participants, assesses a range of phonological awareness skills at syllable and phoneme level and signposts intervention. It was therefore taken forward.

### 3.6 Expert Panel Meeting

Forty panel members attended the online panel meeting and a further five left comments on the same questions using the Padlet (see supplementary material 5). This represents a 32% attrition rate from round one.

#### 3.6.1 COS Agreement

It was agreed by the panel that only outcomes that could be measured with existing assessments should be considered as measures in the COS. For large‐scale data collection to be feasible (MISLToe_SSD phase 2 and 3), any changes to existing practice for speech and language therapy services should represent an improvement on current practice and not place an additional onerous burden on SLTs, children, families or others involved in the assessment process.

No consensus was reached regarding QoL measures. Following discussion about the QoL and generalisation statements it was agreed that, although these are important outcomes, they would not go forward into the COS at this stage. It was acknowledged that SSD may not be an issue that impacts all children's QoL but for some children is a major factor. No panel members were required to collect QoL measures in their routine data collection, although it may be informally collected in discussion with parents and children. Some panel members had used the QoL scales in TOMs (Enderby and John 2019a; 2019b) but few used them routinely except for services that use the RCSLT Online Outcome Tool (ROOT), (Moyse et al., 2020). The FOCUS (Thomas‐Stonell et al., 2013) was occasionally used by some panel members but was considered too long for routine use and often challenging for parents to complete due to high language/literacy demands. Collecting QoL data from children was considered important. The Speech Participation and Activity Assessment of Children (SPAA‐C) (McLeod 2004) had been used by some panel members. It has a version for use with children and is available in seven languages (English, Polish and Turkish are likely to be most relevant in the United Kingdom). The issue of QoL as a routinely measurable COS will be explored more in MISLToe‐SSD phase 2 with the aim of identifying an acceptable and feasible QoL measure to take forward into phase 3. MISLToe‐SSD phase 2 started in autumn 2025 across Wales and England.

The consensus in discussions across the panel members was that generalisation gains could be captured in measures of percentage consonants correct (PCC). Discussion of the other statements led to agreement that several of them described similar outcomes, measured in the same way and could therefore be conflated.

There was full agreement that *Increased speech intelligibility* should be the primary outcome, measured using the ICS (McLeod et al. 2012a), which is completed by parents/caregivers. PCC can also be used as a proxy for intelligibility, if the ICS cannot be completed. In MISLToe‐SSD phase 2, both ICS and PCC will be routinely collected and analysed to evaluate the validity and reliability of the ICS before going forward to a much larger role out of the protocol in MISLToe‐SSD phase 3. There was full agreement on the remaining outcomes and measures, see Table 4.

**TABLE 4:** The final COS.

SSD Outcome	Baseline measure	Outcome measure	Progress measure
Increased speech intelligibility	ICS	ICS	ICS
Increased percentage consonants correct (PCC)	DEAP	DEAP	DEAP
Increased stimulability	DEAP	DEAP	DEAP
Increase in number of phonemes	DEAP	DEAP	DEAP
Increase in percentage vowels correct (PVC)	DEAP	DEAP	DEAP
Increase in percentage phonemes correct (PPC)	DEAP	DEAP	DEAP
Increase in phonological awareness	NAPA	NAPA	NAPA

CAS is a rare but very serious subtype of SSD. The core outcomes for CAS are the same as for other subtypes of SSD, however, different assessment methods are required to capture the nuances of the disorder. It was agreed that the Nuffield Dyspraxia Programme Assessment (Williams and Stephens 2004) would be the recommended assessment for children with CAS, following exploratory assessment for other subtypes of SSD.

#### 3.6.2 Language Measure

Discussion in small groups considered different measures for language disorders that are commonly used by UK SLTs. Several of the expressive language assessments were considered unsuitable for use with children with SSD because their responses can be unintelligible, making it difficult to evaluate the language content accurately. The majority of panel members favoured a sentence repetition task that could act as a screening test for expressive language disorder and as an additional sample of connected speech with known targets, in addition to the sample collected in the DEAP (Dodd et al. 2002). Ideally, these sentences would also elicit a representative sample of phonemes. No existing sets of sentences were identified by panel members. It was therefore agreed that the MISLToe‐SSD research team would investigate existing standardised and informal sentence repetition tasks and include in the diagnostic protocol a set of sentences that would provide information about speech and expressive language.

#### 3.6.3 Minimum Dataset

The discussion about the content of the minimum dataset included the utility of the data, how and when the data should be collected. These data can be collected routinely via online records at each point of contact and can then be extracted in an anonymised format for use in future research. Discussion led to the addition or rewording of common data elements that panel members considered essential to gain a full picture of the episode of care and therefore the effectiveness of the SSD care pathway; see Table 5. It was agreed that the MISLToe‐SSD research team would formulate a definition of an episode of care for use with the COS and minimum dataset.

**TABLE 5:** Final minimum data set.

Common data element
Date of birth
Date of data collection point^*^
Age at data collection point
Sex assigned at birth
Socio‐economic status (postcode)^*^
Total time in intervention (hours.mins)
Have they had previous episode of care before study^*^
Number of episodes of care in study^*^
Duration of session (minutes)
Frequency of session (per week)^*^
Length of episode of care (weeks)
Homework given to anyone between sessions^*^
Location of sessions
Agent of intervention
Goals of intervention
SSD diagnostic label 1^*^
SSD diagnostic label 2^*^
Other diagnostic label (co‐occurrence)^*^
Languages spoken at home
Intervention (named)^*^

*Added or re‐worded following input from Panel.

## 4 Discussion

### 4.1 Summary of Main Findings

The aim of this study was to develop a COS and minimum dataset for future collection of routine data about the speech and language services delivered to children with SSD in the United Kingdom. A modified Delphi process was used with a panel of UK SLTs considered by their peers as specialists or experts in SSD. A previous umbrella review (Harding et al. 2024)^,^ participatory workshops with SLTs (Cleland et al. 2025) and PPIE consultations, including with individuals with lived experience of SSD, formed the basis of the Delphi statements. Consensus was obtained, reducing a long list of 30 potential outcomes to one primary outcome (increased intelligibility) and six secondary outcomes with associated measurement instruments (Table 4). Simultaneous screening of expressive language skills was agreed by 92% of respondents, using a sentence repetition task to be provided by the MISLToe‐SSD team. This would also provide opportunity for a further connected speech sample, in addition to that provided in the DEAP (Masso et al. 2016).

Seventy‐one SLTs representing all four UK devolved countries consented to take part in the Delphi process, with only five (7%) lost to round one. Of the 66 SLTs who took part in round one, 62 (94%) also completed round two, and 45 (68%) took part in the expert panel meeting or contributed online via Padlet. This recruitment and retention compares favourably with similar studies in speech and language therapy (Bishop et al. 2016; Denman et al. 2021; Wallace et al. 2018, 2022) where both clinical and academic SLTs have been recruited.

### 4.2 Strengths

The COMET (Prinsen et al. 2014, 2016; Williamson et al. 2017) recommendations were followed for the modified Delphi process. The long list of outcomes and assessments were developed from an umbrella review of literature (Harding et al. 2024). High levels of consensus were obtained in both rounds with good panel member retention. The MISLToe methodology has been developed so that it can be replicated in other countries and other healthcare systems to develop COS that are aligned with the UK version. Outcomes for SSD are likely to be the same in all languages (e.g., increased intelligibility) although the measures used will differ depending upon the phonetic inventory (consonants and vowels) and phonological rules of the language. This has the potential to facilitate larger scale cross‐linguistic studies into assessment, classification and interventions for SSD.

Current resource issues lead to non‐evidence based assessments being used in clinical practice because they are much cheaper. These include published assessments with no standardisation or basis in research and “informal” screens and assessments made up by clinicians. However, the panel members showed a strong consensus for the use of the DEAP (Dodd et al. 2002), which remains the only comprehensive assessment of SSD that is standardised on a diverse UK population. It was the assessment identified as most suitable for assessing SSD outcomes at baseline, post‐intervention and for tracking progress.

### 4.3 Limitations

The final COS and minimum dataset are intended for use in future research into SSD care pathways delivered by SLTs in the NHS and independent practice in the UK. Data were collected from a small international expert panel, which will be reported separately, with lessons learnt about the difficulty of aligning assessment and intervention protocols in different healthcare systems and in different languages. This differs from other COS which are intended for use in research in different languages and healthcare systems for example, ROMA (Wallace et al. 2018).

It was not possible to include a QoL outcome, despite it being considered as important, as there are no instruments that specifically measure QoL for children with SSD. This has highlighted an opportunity for researchers, clinicians, children, young people and families to develop such a measure. Use of QoL measures by speech and language therapy services, such as TOM and SPAA‐C will be monitored in future iterations of MISLToe‐SSD as it is trialled with UK speech and language therapy services.

The panel identified the DEAP as a means of tracking progress. However, it may not be sensitive enough to track progress for all phonemes, especially over shorter episodes of care. We would suggest in those circumstances that probe word lists are used to provide more accurate and nuanced data for SLTs.

### 4.4 Implications for Practice and Research

The high level of consensus and wider interest in the MISLToe‐SSD study has demonstrated a commitment by SLTs in the United Kingdom for agreed diagnostic terminology and core outcomes. The 2024 SSD Clinical Guidance published by the Royal College of Speech and Language Therapists (RCSLT 2024) utilises the same agreed terminology and definitions.

Gaps in research have been identified in QoL and communicative participation measures for children with SSD. The need for development of cross‐linguistic assessments for SSD was highlighted in the Delphi process as few are currently available for English plus other language bilingual children.

The COS and minimum dataset can be taken forward into MISLToe‐SSD phase two, where an online data collection template will facilitate a pilot study of large‐scale data collection of SSD care pathway outcomes. Identification of the most effective and efficient interventions for different subtypes of SSD will save money for both the NHS and educational settings and improve the life chances of many thousands of children.

## Funding

This project is funded by the National Institute for Health and Care Research (NIHR) under its Research for Patient Benefit (RfPB) Programme (Grant Reference Number NIHR202766). The views expressed are those of the author(s) and not necessarily those of the NIHR or the Department of Health and Social Care.

## Ethics Statement

Ethical approval was provided by Newcastle University HaSS Faculty Ethics Committee (ref 26494/2022)

## Conflicts of Interest

The authors declare no conflicts of interest.

## Supporting information




**Supporting Information**: jlcd70188‐sup‐0001‐SuppMat1COS‐STAR.docx

**Supporting Information**: jlcd70188‐sup‐0002‐SuppMat2Round1survey.pdf

**Supporting Information**: jlcd70188‐sup‐0003‐SuppMat3MinimumDataset.pdf

**Supporting Information**: jlcd70188‐sup‐0004‐SuppMat4Round2survey.pdf

**Supporting Information**: jlcd70188‐sup‐0005‐SuppMat5panel_report.pdf

**Supporting Information**: jlcd70188‐sup‐0006‐SuppMat6Original_Protocol.pdf

## Data Availability

Quantitative data not included in the text is available by application to the lead author.
