# Supplementary material for: Maximising the Impact of Speech and Language Therapy for Children With Speech Sound Disorder (The MISLToe‐SSD) Study: Developing a Core Outcome Set (COS) for Routine Data Collection From UK NHS Speech and Language Therapy Services
Source: Int J Lang Commun Disord. 2026 Jan 9;61(1):e70188. doi: 10.1111/1460-6984.70188 (PMC12784794; doi:10.1111/1460-6984.70188)
Supplement: Supplementary file 1 — Supporting Information: jlcd70188‐sup‐0001‐SuppMat1COS‐STAR.docx [file JLCD-61-0-s002.docx]

**SUPPLEMENTARY MATERIAL 1**

**Core Outcome Set-STandards for Reporting: The COS-STAR Statement Checklist for**

**Maximising the Impact of Speech and Language Therapy for children with Speech Sound Disorder (The MISLToe-SSD) Study: Developing a Core Outcome Set (COS) through a modified Delphi process**

**[page numbers as per submitted manuscript]**

| **SECTION/TOPIC** | **ITEM No.** | **CHECKLIST ITEM** | **REPORTED ON PAGE NUMBER** |
| --- | --- | --- | --- |
| TITLE/ABSTRACT | | | |
| Title | 1a | Identify in the title that the paper reports the development of a COS | 1 |
| Abstract | 1b | Provide a structured summary | 2 |
| INTRODUCTION | | | |
| Background and Objectives | 2a | Describe the background and explain the rationale for developing the COS. | 3 |
|  | 2b | Describe the specific objectives with reference to developing a COS. | 3 |
| Scope | 3a | Describe the health condition(s) and population(s) covered by the COS. | 3, 4 |
|  | 3b | Describe the intervention(s) covered by the COS. | 3, 4 |
|  | 3c | Describe the setting(s) in which the COS is to be applied. | 3, 4 |
| METHODS | | | |
| Protocol/Registry Entry | 4 | Indicate where the COS development protocol can be accessed, if available, and/or the study registration details. | 4 |
| Participants | 5 | Describe the rationale for stakeholder groups involved in the COS development process, eligibility criteria for participants from each group, and a description of how the individuals involved were identified. | 4 |
| Information Sources | 6a | Describe the information sources used to identify an initial list of outcomes. | 6 |
|  | 6b | Describe how outcomes were dropped/combined, with reasons (if applicable). | n/a |
| Consensus Process | 7 | Describe how the consensus process was undertaken. | 6 |
| Outcome Scoring | 8 | Describe how outcomes were scored and how scores were summarised. | 6, 7 |
| Consensus Definition | 9a | Describe the consensus definition. | 7, 8 |
|  | 9b | Describe the procedure for determining how outcomes were included or excluded from consideration during the consensus process. | 7 |
| Ethics and Consent | 10 | Provide a statement regarding the ethics and consent issues for the study. | 4, 8 |
| RESULTS | | | |
| Protocol Deviations | 11 | Describe any changes from the protocol (if applicable), with reasons, and describe what impact these changes have on the results. | 9 |
| Participants | 12 | Present data on the number and relevant characteristics of the people involved at all stages of COS development. | 10 |
| Outcomes | 13a | List all outcomes considered at the start of the consensus process. | 12 |
|  | 13b | Describe any new outcomes introduced and any outcomes dropped, with reasons, during the consensus process. | 11, 12 |
| COS | 14 | List the outcomes in the final COS. | 15 |
| DISCUSSION | | | |
| Limitations | 15 | Discuss any limitations in the COS development process. | 17 |
| Conclusions | 16 | Provide an interpretation of the final COS in the context of other evidence, and implications for future research. | 17 |
| OTHER INFORMATION | | | |
| Funding | 17 | Describe sources of funding/role of funders. | 18 |
| Conflicts of Interest | 18 | Describe any conflicts of interest within the study team and how these were managed. | 18 |

*From: Kirkham JJ, Gorst S, Altman DG, Blazeby JM, Clarke M, Devane D, et al. (2016) Core Outcome Set–STAndards for Reporting: The COS-STAR Statement. PLoS Med 13(10): e1002148. https://doi.org/10.1371/journal.pmed.1002148*
