# Supplementary material for: Maximising the Impact of Speech and Language Therapy for Children With Speech Sound Disorder (The MISLToe‐SSD) Study: Developing a Core Outcome Set (COS) for Routine Data Collection From UK NHS Speech and Language Therapy Services
Source: Int J Lang Commun Disord. 2026 Jan 9;61(1):e70188. doi: 10.1111/1460-6984.70188 (PMC12784794; doi:10.1111/1460-6984.70188)
Supplement: Supplementary file 2 — Supporting Information: jlcd70188‐sup‐0002‐SuppMat2Round1survey.pdf [file JLCD-61-0-s004.pdf]

MISLToe-SSD UK Delphi Round 1

Welcome to Round 1 of the MISLToe-SSD Delphi process

Maximising the **I**mpact of **S**peech and **L**anguage **T**herapy for children with **S**peech **S**ound **D**isorder (The MISLToe-SSD Study)

Maximising the **I**mpact of **S**peech and **L**anguage **T**herapy for children with **S**peech **S**ound **D**isorder (The MISLToe-SSD Study)

You can Click here to visit our website and follow us on Twitter @MISLToe_SSD

Thank you for agreeing to be a member of the MISLToe-SSD expert panel. The aim of our work together is to reach a consensus agreement on a core set of outcomes from speech and language therapy (SLT) intervention for children with speech sound disorders (SSD). In addition to the outcomes of intervention, we aim to gain consensus on the way outcomes are assessed.

Welcome to the first round of the Delphi process. In this round we will ask you to:

- judge a series of statements about SSD outcomes,
- give a rationale for some of your choices,
- reduce a long list of assessments for SSD to a shorter list that will go forward for more detailed judgement in Round 2,
- answer some questions about the minimum dataset required to support collection in practice of the outcome data.

Following this round we will provide feedback about how your answers compared to other anonymised panel members. You will have an opportunity to tell us if your thinking has changed or if you forgot to mention something. The results of the revised round one will inform the content of round two.

We hope you enjoy taking part in this process and look forward to continuing this journey with you.

This Delphi round will take you about 45 minutes.There are 3 sections to this round: outcomes, assessments (outcome measures) and the minimum dataset.

You can save your contribution and return at any time. Please submit this survey by midnight on Friday 17th February or your contribution will not be counted.

Outcomes

The following statements relate to the outcomes of interventions for SSD. The outcomes included here are all taken from intervention studies reported in peer reviewed journals identified in an umbrella review (a review of systematic reviews). Some of these outcomes may be unfamiliar to you or worded in an unusual way. At this stage we have tried to provide a long and inclusive list. Please consider them in relation to your clinical work, teaching and research.

The outcome is the ultimate or long term goal of one or more episodes of intervention for a child with SSD. For each outcome we will ask how important you think it is as an outcome of intervention for SSD. We will also ask you whether you think it should be considered as a primary outcome of intervention. Primary outcomes are the ones of most importance for clients and their families and/or speech and language therapists/pathologists (SLT/Ps). Secondary outcomes evaluate other beneficial effects of the intervention or explain additional effects of the intervention.

Population: Unless stated otherwise, please assume that we are considering the wider population of children who have received intervention from SLT/Ps for any and all subtypes of SSD of unknown origin. At this stage we are considering all of these children, so if an outcome is very important for a few children with SSD please rate with the same importance as an outcome that is very important for most of the children.

Timescale: Please remember that these are the desired outcomes at the end of one or more episodes of care, not for individual sessions.

What is your name? *Optional*

What is your email address? *Optional*

Please enter a valid email address.

Rating the importance of SSD intervention outcomes

The following statements are about the importance of different outcomes following intervention for SSD for the population and timescale mentioned above. Some of these outcomes may mean the same as other, differently worded outcomes or may be similar, although not identical, to other outcomes. One aim of this section is to reduce the list, so your rating of each item is important to us. Please rate the importance of the following statements as an outcome of intervention for SSD on the 5 point scale. For the statements you have rated 1 or 5 please explain why. You can also leave comments against any other statement if you wish to.

	✱ *Required*					
	1. Not at all important	2. Slightly important	3. Moderately important	4. Very important	5. Essential	Please explain your rating *Optional*
Increased speech intelligibility	☐	☐	☐	☐	☐	
Increased confidence when talking	☐	☐	☐	☐	☐	
Improved quality of life	☐	☐	☐	☐	☐	
Improved communicative activity and participation	☐	☐	☐	☐	☐	
Improved language	☐	☐	☐	☐	☐	
Improved vocabulary	☐	☐	☐	☐	☐	

Increased Percentage Consonants Correct (PCC)	☐	☐	☐	☐	☐	
Increase in Percentage Phonemes Correct (PPC)	☐	☐	☐	☐	☐	
Increase in Percentage Vowels Correct (PVC)	☐	☐	☐	☐	☐	
Increase in Percentage of Words Correct (PWC)	☐	☐	☐	☐	☐	
Increase in Percentage of Intelligible Utterances (PIU)	☐	☐	☐	☐	☐	
Decrease in Proportion of Errors (PoE)	☐	☐	☐	☐	☐	
Decrease in phonological variability	☐	☐	☐	☐	☐	
Increased accuracy of target	☐	☐	☐	☐	☐	

Increase in production of target sounds	☐	☐	☐	☐	☐	
Increase in phonological awareness	☐	☐	☐	☐	☐	
Increased stimulability	☐	☐	☐	☐	☐	
Improved oromotor skills	☐	☐	☐	☐	☐	
Increase in number of phonemes	☐	☐	☐	☐	☐	
Increase in egressive output	☐	☐	☐	☐	☐	
Generalisation across linguistic units	☐	☐	☐	☐	☐	
Generalisation across word position	☐	☐	☐	☐	☐	

Generalisation to a new context	☐	☐	☐	☐	☐	
Generalisation of known sounds	☐	☐	☐	☐	☐	
Generalisation of the intervention target	☐	☐	☐	☐	☐	
Generalisation related to the target (e.g., generalisation to other phonemes within and across sound classes)	☐	☐	☐	☐	☐	
Increased mean length of utterance (MLU)	☐	☐	☐	☐	☐	
Increase in percentage child utterance attempts that are fully intelligible from language sample	☐	☐	☐	☐	☐	
Parent report on increased structural complexity	☐	☐	☐	☐	☐	

Parent report on increased phrase complexity	☐	☐	☐	☐	☐	

Primary and secondary outcomes

Are some outcomes more important than others?
A **primary outcome** is an outcome that everyone will agree has great importance and will be a desired goal in most interventions. These may be different for children and families compared to those that speech and language therapists consider primary outcomes. There may be more than one primary outcome if several outcomes are equally important. Outcomes that are not primary will automatically be secondary outcomes. In this next section please make a judgement as to whether the outcomes might be a primary outcome for children and families and/or for speech and language therapists.

Please tell us which outcomes could be considered as primary outcomes for SSD interventions ✱ *Required*

Please don't select more than 2 answer(s) per row.

Please select at least 30 answer(s).

	Children/Families Primary outcome	SLT Primary outcome	Neither (i.e. secondary or other outcome)
Increased speech intelligibility	☐	☐	☐
Increased confidence when talking	☐	☐	☐
Improved quality of life	☐	☐	☐
Improved communicative activity and participation	☐	☐	☐
Improved language	☐	☐	☐
Improved vocabulary	☐	☐	☐
Increased Percentage Consonants Correct (PCC)	☐	☐	☐
Increase in Percentage Phonemes Correct (PPC)	☐	☐	☐
Increase in Percentage Vowels Correct (PVC)	☐	☐	☐
Increase in Percentage of Words Correct (PWC)	☐	☐	☐
Increase in Percentage of Intelligible Utterances (PIU)	☐	☐	☐
Decrease in Proportion of Errors (PoE)	☐	☐	☐
Decrease in phonological variability	☐	☐	☐
Increased accuracy of target	☐	☐	☐
Increase in production of target sounds	☐	☐	☐
Increase in phonological awareness	☐	☐	☐
Increased stimulability	☐	☐	☐
Improved oromotor skills	☐	☐	☐
Increase in number of phonemes	☐	☐	☐
Increase in egressive output	☐	☐	☐
Generalisation across linguistic units	☐	☐	☐
Generalisation across word position	☐	☐	☐
Generalisation to a new context	☐	☐	☐
Generalisation of known sounds	☐	☐	☐
Generalisation of the intervention target	☐	☐	☐

Generalisation related to the target (e.g., generalisation to other phonemes within and across sound classes)	☐	☐	☐
Increased mean length of utterance (MLU)	☐	☐	☐
Increase in percentage child utterance attempts that are fully intelligible from language sample	☐	☐	☐
Parent report on increased structural complexity	☐	☐	☐
Parent report on increased phrase complexity	☐	☐	☐

Please tell us why you chose the primary outcomes that you did. *Optional*

Outcomes: What have we missed?

Are there any important outcomes of intervention for SSD that are not listed above.

Assessments: How we measure the outcomes

In this next section, we will ask about your experience and views on a range of different assessments that can be used with children with suspected SSD/SSD of unknown origin by SLT/Ps. These assessments were all identified in the umbrella review mentioned above.

For children referred to SLT/P services with suspected SSD, please answer the following questions about assessments that are used to measure outcomes. When we ask about assessmentsthat you know, we mean that you have some knowledge or familaiarity with the assessment, not just that you have heard of it.

Considering the assessment: Arizona Articulation Proficiency Scale (AAPS)

Considering the assessment: Arizona Articulation Proficiency Scale (AAPS) Do you know this assessment? ✱ *Required*

- ○ Yes
- ○ No

Please add a bit more information

Considering the assessment: Arizona Articulation Proficiency Scale (AAPS)

	✱ *Required*		
	Yes	No	Don't know
Have you used this assessment?	◯	◯	◯
Would you use this if it was available?	◯	◯	◯
Is this assessment suitable for baseline assessment?	◯	◯	◯
Is this assessment suitable for outcome assessment?	◯	◯	◯
Is this assessment suitable for progress assessment?	◯	◯	◯

Do you have any information to add about this assessment? *Optional*

Considering the assessment: Bankson-Bernthal Test of Phonology (BBTOP)

Considering the assessment: Bankson-Bernthal Test of Phonology (BBTOP) Do you know this assessment? ✱ *Required*

- ○ Yes
- ○ No

Please add a bit more information

Considering the assessment: Bankson-Bernthal Test of Phonology (BBTOP)

	✱ *Required*	
	Yes	No
Have you used this assessment?	○	○
Would you use this if it was available?	○	○
Is this assessment suitable for baseline assessment?	○	○
Is this assessment suitable for outcome assessment?	○	○
Is this assessment suitable for progress assessment?	○	○

Do you have any information to add about this assessment? *Optional*

Considering the assessment: Children’s Test of Nonword Repetition (Gathercole)

Considering the assessment: Children’s Test of Nonword Repetition (Gathercole) Do you know this assessment? ✱ *Required*

- ○ Yes
- ○ No

Please add a bit more information

Considering the assessment: Children's Test of Nonword Repetition (Gathercole)

	✱ *Required*	
	Yes	No
Have you used this assessment?	○	○
Would you use this if it was available?	○	○
Is this assessment suitable for baseline assessment?	○	○
Is this assessment suitable for outcome assessment?	○	○
Is this assessment suitable for progress assessment?	○	○

Do you have any information to add about this assessment? *Optional*

Considering the assessment: Comprehensive Test of Phonological Processing – second edition (CTOPP-2)

Considering the assessment: Comprehensive Test of Phonological Processing – second edition (CTOPP-2) Do you know this assessment? ✱ *Required*

- ○ Yes
- ○ No

Please add a bit more information

Considering the assessment: Comprehensive Test of Phonological Processing – second edition (CTOPP-2)

	✱ *Required*	
	Yes	No
Have you used this assessment?	○	○
Would you use this if it was available?	○	○
Is this assessment suitable for baseline assessment?	○	○
Is this assessment suitable for outcome assessment?	○	○
Is this assessment suitable for progress assessment?	○	○

Do you have any information to add about this assessment? *Optional*

Considering the assessment: Comprehensive Test of Phonological Processing and Print Processing

Considering the assessment: Comprehensive Test of Phonological Processing and Print Processing Do you know this assessment? ✱ *Required*

- ◯ Yes
- ◯ No

Please add a bit more information

Considering the assessment: Comprehensive Test of Phonological Processing and Print Processing

	✱ *Required*	
	Yes	No
Have you used this assessment?	◯	◯
Would you use this if it was available?	◯	◯
Is this assessment suitable for baseline assessment?	◯	◯
Is this assessment suitable for outcome assessment?	◯	◯
Is this assessment suitable for progress assessment?	◯	◯

Do you have any information to add about this assessment? *Optional*

Considering the assessment: Computer-Based Phonological Awareness Assessment

Considering the assessment: Computer-Based Phonological Awareness Assessment Do you know this assessment? ✱
Required

- ◯ Yes
- ◯ No

Please add a bit more information

Considering the assessment: Computer-Based Phonological Awareness Assessment

	✱ *Required*	
	Yes	No
Have you used this assessment?	○	○
Would you use this if it was available?	○	○
Is this assessment suitable for baseline assessment?	○	○
Is this assessment suitable for outcome assessment?	○	○
Is this assessment suitable for progress assessment?	○	○

Do you have any information to add about this assessment? *Optional*

Considering the assessment: Computerized Articulation and Phonology Evaluation System (CAPES)

Considering the assessment: Computerized Articulation and Phonology Evaluation System (CAPES) Do you know this assessment? ✱ *Required*

- Yes
- No

Please add a bit more information

Considering the assessment: Computerized Articulation and Phonology Evaluation System (CAPES)

	✱ *Required*	
	Yes	No
Have you used this assessment?	○	○
Would you use this if it was available?	○	○
Is this assessment suitable for baseline assessment?	○	○
Is this assessment suitable for outcome assessment?	○	○
Is this assessment suitable for progress assessment?	○	○

Do you have any information to add about this assessment? *Optional*

Considering the assessment: Denver Articulation Screening Exam

Considering the assessment: Denver Articulation Screening Exam Do you know this assessment? ✱ *Required*

- ○ Yes
- ○ No

Please add a bit more information

Considering the assessment: Denver Articulation Screening Exam

	✱ *Required*	
	Yes	No
Have you used this assessment?	○	○
Would you use this if it was available?	○	○
Is this assessment suitable for baseline assessment?	○	○
Is this assessment suitable for outcome assessment?	○	○
Is this assessment suitable for progress assessment?	○	○

Do you have any information to add about this assessment? *Optional*

Considering the assessment: Diagnostic Evaluation of Articulation and Phonology (DEAP)

Considering the assessment: Diagnostic Evaluation of Articulation and Phonology (DEAP) Do you know this assessment? *
Required

- Yes
- No

Please add a bit more information

Considering the assessment: Diagnostic Evaluation of Articulation and Phonology (DEAP)

	✱ *Required*	
	Yes	No
Have you used this assessment?	○	○
Would you use this if it was available?	○	○
Is this assessment suitable for baseline assessment?	○	○
Is this assessment suitable for outcome assessment?	○	○
Is this assessment suitable for progress assessment?	○	○

Do you have any information to add about this assessment? *Optional*

Considering the assessment: Edinburgh Articulation Test (EAT)

Considering the assessment: Edinburgh Articulation Test (EAT) Do you know this assessment? ✱ *Required*

- Yes
- No

Please add a bit more information

Considering the assessment: Edinburgh Articulation Test (EAT)

	✱ *Required*	
	Yes	No
Have you used this assessment?	○	○
Would you use this if it was available?	○	○
Is this assessment suitable for baseline assessment?	○	○
Is this assessment suitable for outcome assessment?	○	○
Is this assessment suitable for progress assessment?	○	○

Do you have any information to add about this assessment? *Optional*

Considering the assessment: Goldman-Fristoe Test of Articulation (GFTA)

Considering the assessment: Goldman-Fristoe Test of Articulation (GFTA) Do you know this assessment? ✱ *Required*

- Yes
- No

Please add a bit more information

Considering the assessment: Goldman-Fristoe Test of Articulation (GFTA)

	✱ *Required*	
	Yes	No
Have you used this assessment?	○	○
Would you use this if it was available?	○	○
Is this assessment suitable for baseline assessment?	○	○
Is this assessment suitable for outcome assessment?	○	○
Is this assessment suitable for progress assessment?	○	○

Do you have any information to add about this assessment? *Optional*

Considering the assessment: Glaspey Dynamic Assessment of Phonology (GDAP)

Considering the assessment: Glaspey Dynamic Assessment of Phonology (GDAP) Do you know this assessment? ✱ *Required*

- ○ Yes
- ○ No

Please add a bit more information

Considering the assessment: Glaspey Dynamic Assessment of Phonology (GDAP)

	✱ *Required*	
	Yes	No
Have you used this assessment?	○	○
Would you use this if it was available?	○	○
Is this assessment suitable for baseline assessment?	○	○
Is this assessment suitable for outcome assessment?	○	○
Is this assessment suitable for progress assessment?	○	○

Do you have any information to add about this assessment? *Optional*

Considering the assessment: Grammar and Phonology Screening (GAPS)

Considering the assessment: Grammar and Phonology Screening (GAPS) Do you know this assessment? ✱ *Required*

- ○ Yes
- ○ No

Please add a bit more information

Considering the assessment: Grammar and Phonology Screening (GAPS)

	✱ *Required*	
	Yes	No
Have you used this assessment?	○	○
Would you use this if it was available?	○	○
Is this assessment suitable for baseline assessment?	○	○
Is this assessment suitable for outcome assessment?	○	○
Is this assessment suitable for progress assessment?	○	○

Do you have any information to add about this assessment? *Optional*

Considering the assessment: Hodson Assessment of Phonological Patterns-3 (HAPP-3)

Considering the assessment: Hodson Assessment of Phonological Patterns-3 (HAPP-3) Do you know this assessment? ✱
Required

- Yes
- No

Please add a bit more information

Considering the assessment: Hodson Assessment of Phonological Patterns-3 (HAPP-3)

	✱ *Required*	
	Yes	No
Have you used this assessment?	○	○
Would you use this if it was available?	○	○
Is this assessment suitable for baseline assessment?	○	○
Is this assessment suitable for outcome assessment?	○	○
Is this assessment suitable for progress assessment?	○	○

Do you have any information to add about this assessment? *Optional*

Considering the assessment: Individual Growth Development Indicator: Rhyming.

Considering the assessment: *Individual Growth Development Indicator: Rhyming. Do you know this asessment?* ✱ *Required*

- ○ Yes
- ○ No

Please add a bit more information

Considering the assessment: *Individual Growth Development Indicator: Rhyming*

	✱ *Required*	
	Yes	No
Have you used this assessment?	○	○
Would you use this if it was available?	○	○
Is this assessment suitable for baseline assessment?	○	○
Is this assessment suitable for outcome assessment?	○	○
Is this assessment suitable for progress assessment?	○	○

Do you have any information to add about this assessment? *Optional*

Considering the assessment: Preschool and Primary Inventory of Phonological Awareness (PIPA)

Considering the assessment: Preschool and Primary Inventory of Phonological Awareness (PIPA) Do you know this asessment?
✱ *Required*

- Yes
- No

Please add a bit more information

Considering the assessment: Preschool and Primary Inventory of Phonological Awareness (PIPA)

	✱ *Required*	
	Yes	No
Have you used this assessment?	○	○
Would you use this if it was available?	○	○
Is this assessment suitable for baseline assessment?	○	○
Is this assessment suitable for outcome assessment?	○	○
Is this assessment suitable for progress assessment?	○	○

Do you have any information to add about this assessment? *Optional*

Considering the assessment: McDonald Screening Deep Test of Articulation

Considering the assessment: McDonald Screening Deep Test of Articulation Do you know this asessment? ✱ *Required*

- ○ Yes
- ○ No

Please add a bit more information

Considering the assessment: McDonald Screening Deep Test of Articulation

	✱ *Required*	
	Yes	No
Have you used this assessment?	○	○
Would you use this if it was available?	○	○
Is this assessment suitable for baseline assessment?	○	○
Is this assessment suitable for outcome assessment?	○	○
Is this assessment suitable for progress assessment?	○	○

Do you have any information to add about this assessment? *Optional*

Considering the assessment: Oral speech mechanism screen examination (OSMSE)

Considering the assessment: Oral speech mechanism screen examination (OSMSE) Do you know this asessment? ✱ *Required*

- ○ Yes
- ○ No

Please add a bit more information

Considering the assessment: Oral speech mechanism screen examination (OSMSE)

	✱ *Required*	
	Yes	No
Have you used this assessment?	○	○
Would you use this if it was available?	○	○
Is this assessment suitable for baseline assessment?	○	○
Is this assessment suitable for outcome assessment?	○	○
Is this assessment suitable for progress assessment?	○	○

Do you have any information to add about this assessment? *Optional*

Considering the assessment: Phonological Abilities Test (Muter)

Considering the assessment: Phonological Abilities Test (Muter) Do you know this asessment? ✱ *Required*

- ○ Yes
- ○ No

Please add a bit more information

Considering the assessment: Phonological Abilities Test (Muter)

	✱ *Required*	
	Yes	No
Have you used this assessment?	○	○
Would you use this if it was available?	○	○
Is this assessment suitable for baseline assessment?	○	○
Is this assessment suitable for outcome assessment?	○	○
Is this assessment suitable for progress assessment?	○	○

Do you have any information to add about this assessment? *Optional*

Considering the assessment: Phonological Assessment Battery (PhAB)

Considering the assessment: Phonological Assessment Battery (PhAB) Do you know this asessment? ✱ *Required*

- ○ Yes
- ○ No

Please add a bit more information

Considering the assessment: Phonological Assessment Battery (PhAB)

	✱ *Required*	
	Yes	No
Have you used this assessment?	○	○
Would you use this if it was available?	○	○
Is this assessment suitable for baseline assessment?	○	○
Is this assessment suitable for outcome assessment?	○	○
Is this assessment suitable for progress assessment?	○	○

Do you have any information to add about this assessment? *Optional*

Considering the assessment: Phonological Assessment of Child Speech (PACS)

Considering the assessment: Phonological Assessment of Child Speech (PACS) Do you know this asessment? ✱ *Required*

- ○ Yes
- ○ No

Please add a bit more information

Considering the assessment: Phonological Assessment of Child Speech (PACS)

	✱ *Required*	
	Yes	No
Have you used this assessment?	○	○
Would you use this if it was available?	○	○
Is this assessment suitable for baseline assessment?	○	○
Is this assessment suitable for outcome assessment?	○	○
Is this assessment suitable for progress assessment?	○	○

Do you have any information to add about this assessment? *Optional*

Considering the assessment: Phonological Awareness Literacy Screening– PreK (PALS-PreK)

Considering the assessment: Phonological Awareness Literacy Screening– PreK (PALS-PreK) Do you know this asessment? ✱
Required

- ○ Yes
- ○ No

Please add a bit more information

Considering the assessment: Phonological Awareness Literacy Screening– PreK (PALS-PreK)

	✱ *Required*	
	Yes	No
Have you used this assessment?	○	○
Would you use this if it was available?	○	○
Is this assessment suitable for baseline assessment?	○	○
Is this assessment suitable for outcome assessment?	○	○
Is this assessment suitable for progress assessment?	○	○

Do you have any information to add about this assessment? *Optional*

Considering the assessment: Phonological Awareness Test (Robertson and Salter)

Considering the assessment: Phonological Awareness Test (Robertson and Salter) Do you know this asessment? ✱ *Required*

- ◯ Yes
- ◯ No

Please add a bit more information

Considering the assessment: Phonological Awareness Test (Robertson and Salter)

	✱ *Required*	
	Yes	No
Have you used this assessment?	○	○
Would you use this if it was available?	○	○
Is this assessment suitable for baseline assessment?	○	○
Is this assessment suitable for outcome assessment?	○	○
Is this assessment suitable for progress assessment?	○	○

Do you have any information to add about this assessment? *Optional*

Considering the assessment: Phonological Knowledge Protocol (PKP)

Considering the assessment: Phonological Knowledge Protocol (PKP) Do you know this asessment? ✱ *Required*

- ○ Yes
- ○ No

Please add a bit more information

Considering the assessment: Phonological Knowledge Protocol (PKP)

✱ *Required*	Yes	No
Have you used this assessment?	○	○
Would you use this if it was available?	○	○
Is this assessment suitable for baseline assessment?	○	○
Is this assessment suitable for outcome assessment?	○	○
Is this assessment suitable for progress assessment?	○	○

Do you have any information to add about this assessment? *Optional*

Considering the assessment: Phonological Variability Test

Considering the assessment: Phonological Variability Test Do you know this asessment? ✱ *Required*

- ○ Yes
- ○ No

Please add a bit more information

Considering the assessment: Phonological Variability Test

	✱ *Required*	
	Yes	No
Have you used this assessment?	○	○
Would you use this if it was available?	○	○
Is this assessment suitable for baseline assessment?	○	○
Is this assessment suitable for outcome assessment?	○	○
Is this assessment suitable for progress assessment?	○	○

Do you have any information to add about this assessment? *Optional*

Considering the assessment: Scaffolding Scale of Stimulability (SSS)

Considering the assessment: Scaffolding Scale of Stimulability (SSS) Do you know this asessment? ✱ *Required*

- ○ Yes
- ○ No

Please add a bit more information

Considering the assessment: Scaffolding Scale of Stimulability (SSS)

	✱ *Required*	
	Yes	No
Have you used this assessment?	○	○
Would you use this if it was available?	○	○
Is this assessment suitable for baseline assessment?	○	○
Is this assessment suitable for outcome assessment?	○	○
Is this assessment suitable for progress assessment?	○	○

Do you have any information to add about this assessment? *Optional*

Considering the assessment: School Speech Questionnaire (Bergman)

Considering the assessment: School Speech Questionnaire (Bergman) Do you know this asessment? ✱ *Required*

- ◯ Yes
- ◯ No

Please add a bit more information

Considering the assessment: School Speech Questionnaire (Bergman)

	✱ *Required*	
	Yes	No
Have you used this assessment?	○	○
Would you use this if it was available?	○	○
Is this assessment suitable for baseline assessment?	○	○
Is this assessment suitable for outcome assessment?	○	○
Is this assessment suitable for progress assessment?	○	○

Do you have any information to add about this assessment? *Optional*

Considering the assessment: Sutherland Phonological Awareness Test–Revised

Considering the assessment: Sutherland Phonological Awareness Test–Revised Do you know this asessment? ✱ *Required*

- ○ Yes
- ○ No

Please add a bit more information

Considering the assessment: Sutherland Phonological Awareness Test–Revised

	✱ *Required*	
	Yes	No
Have you used this assessment?	○	○
Would you use this if it was available?	○	○
Is this assessment suitable for baseline assessment?	○	○
Is this assessment suitable for outcome assessment?	○	○
Is this assessment suitable for progress assessment?	○	○

Do you have any information to add about this assessment? *Optional*

Considering the assessment: Syllable Repetition Task (Shriberg et al.)

Considering the assessment: Syllable Repetition Task (Shriberg et al.) Do you know this asessment? ✱ *Required*

- ○ Yes
- ○ No

Please add a bit more information

Considering the assessment: Syllable Repetition Task (Shriberg et al.)

	✱ *Required*	
	Yes	No
Have you used this assessment?	◯	◯
Would you use this if it was available?	◯	◯
Is this assessment suitable for baseline assessment?	◯	◯
Is this assessment suitable for outcome assessment?	◯	◯
Is this assessment suitable for progress assessment?	◯	◯

Do you have any information to add about this assessment? *Optional*

Considering the assessment: Test of Phonological Awareness–Second Edition: Plus Test of Preschool Early Literacy (TOPA-2+)

Considering the assessment: Test of Phonological Awareness–Second Edition: Plus Test of Preschool Early Literacy (TOPA-2+) Do you know this asessment? ✱ *Required*

- ○ Yes
- ○ No

Please add a bit more information

Considering the assessment: Test of Phonological Awareness–Second Edition: Plus Test of Preschool Early Literacy (TOPA-2+)

	✱ *Required*	
	Yes	No
Have you used this assessment?	○	○
Would you use this if it was available?	○	○
Is this assessment suitable for baseline assessment?	○	○
Is this assessment suitable for outcome assessment?	○	○
Is this assessment suitable for progress assessment?	○	○

Do you have any information to add about this assessment? *Optional*

Considering the assessment: Templin-Darley Articulation Screening Test

Considering the assessment: Templin-Darley Articulation Screening Test Do you know this asessment? ✱ *Required*

- ○ Yes
- ○ No

Please add a bit more information

Considering the assessment: Templin-Darley Articulation Screening Test

	✱ *Required*	
	Yes	No
Have you used this assessment?	○	○
Would you use this if it was available?	○	○
Is this assessment suitable for baseline assessment?	○	○
Is this assessment suitable for outcome assessment?	○	○
Is this assessment suitable for progress assessment?	○	○

Do you have any information to add about this assessment? *Optional*

Considering the assessment: Test of polysyllables (Gozzard et al.)

Considering the assessment: Test of polysyllables (Gozzard et al.) Do you know this asessment? ✱ *Required*

- ○ Yes
- ○ No

Please add a bit more information

Considering the assessment: Test of polysyllables (Gozzard et al.)

	✱ *Required*	
	Yes	No
Have you used this assessment?	○	○
Would you use this if it was available?	○	○
Is this assessment suitable for baseline assessment?	○	○
Is this assessment suitable for outcome assessment?	○	○
Is this assessment suitable for progress assessment?	○	○

Do you have any information to add about this assessment? *Optional*

Considering the assessment: Verbal Motor Production Assessment for Children (Hayden and Square)

Considering the assessment: Verbal Motor Production Assessment for Children (Hayden and Square) Do you know this asessment? ✱ *Required*

- ○ Yes
- ○ No

Please add a bit more information

Considering the assessment: Verbal Motor Production Assessment for Children (Hayden and Square)

	✱ *Required*	
	Yes	No
Have you used this assessment?	○	○
Would you use this if it was available?	○	○
Is this assessment suitable for baseline assessment?	○	○
Is this assessment suitable for outcome assessment?	○	○
Is this assessment suitable for progress assessment?	○	○

Do you have any information to add about this assessment? *Optional*

Considering the assessment: Word Complexity Measure (Stoel-Gammon)

Considering the assessment: Word Complexity Measure (Stoel-Gammon) Do you know this asessment? ✱ *Required*

- ◯ Yes
- ◯ No

Please add a bit more information

Considering the assessment: Word Complexity Measure (Stoel-Gammon)

	✱ *Required*	
	Yes	No
Have you used this assessment?	○	○
Would you use this if it was available?	○	○
Is this assessment suitable for baseline assessment?	○	○
Is this assessment suitable for outcome assessment?	○	○
Is this assessment suitable for progress assessment?	○	○

Do you have any information to add about this assessment? *Optional*

A little bit more about assessments: Have we missed any?

Have we missed any assessments? Which assessments, not listed above, do you use to assess children with SSD? This can include assessments used for assessing any aspect of the child's speech. Please give the name of the assessment, what it assesses and when you would use it (e.g. baseline/progress/outcome). If you have nothing to add, please write 'none'. ✱ *Required*

When assessing a child with SSD, do you routinely assess their language skills? ✱ *Required*

- ☐ Yes, in depth
- ☐ Yes, a screen
- ☐ Only if language delay or disorder is flagged
- ☐ No

Please list your two most frequently used language assessments for children with a primary SSD

Are there adverse effects of assessment or intervention?

We are concentrating on beneficial outcomes of intervention for SSD. Do you think we should also consider any harmful effects or negative outcomes? These might be if inadequate assessment leads to an incorrect diagosis; if the intervention is provided with reduced dosage; if the intervention provided is known to be ineffective for the given diagnosis or there are more effective interventions that have not been provided. ✱ *Required*

- Yes
- No

Please tell us what harmful effects of SSD intervention you think there could be?

The Minimum Dataset

The minimum dataset is a list of routinely collected common data elements that add value and help us make sense of the data from the outcome measures. These are things like age, type of intervention, distribution and length of sessions. For each of these it is important that they are recorded identically so that the data can be processed quickly and easily.

In the next section we will ask you a series of yes/no questions about what you think is important to collect in the minimum dataset. We will also ask about your (or your clinical service's) ability to collect each type of data.

	Is this important to make sense of outcome data? ✱ *Required*	Do you routinely record this? ✱ *Required*
Date of birth	Please select	Please select
Age at data collection point	Please select	Please select
Sex assigned at birth	Please select	Please select
Socio-economic status	Please select	Please select
Duration of session (minutes)	Please select	Please select
Spacing of session (per week)	Please select	Please select
Length of episode of care (weeks)	Please select	Please select
Total time in intervention (hours.mins)	Please select	Please select
Homework given	Please select	Please select
Location of sessions	Please select	Please select
Agent of intervention	Please select	Please select
Goals of intervention	Please select	Please select
Number of episodes of care	Please select	Please select
Diagnostic label 1	Please select	Please select
Diagnostic label 2	Please select	Please select
Birth order	Please select	Please select
Number of siblings	Please select	Please select
Languages spoken at home	Please select	Please select

If you work in clinical services, which clinical data collection system does your service use? E.g. SystemOne

Thank you!

Maximising the Impact of Speech and Language Therapy for children with Speech Sound Disorder (The MISLToe-SSD Study)

Thank you for completing Round 1 of the MISLToe-SSD Delphi. We will be in touch soon with the results.

Key for selection options

79.1.a - Is this important to make sense of outcome data?
Yes
No

79.1.b - Do you routinely record this?
Yes
No

79.2.a - Is this important to make sense of outcome data?
Yes
No

79.2.b - Do you routinely record this?
Yes
No

79.3.a - Is this important to make sense of outcome data?
Yes
No

79.3.b - Do you routinely record this?
Yes
No

79.4.a - Is this important to make sense of outcome data?
Yes
No

79.4.b - Do you routinely record this?
Yes
No

79.5.a - Is this important to make sense of outcome data?
Yes
No

79.5.b - Do you routinely record this?

Yes
No

79.6.a - Is this important to make sense of outcome data?
Yes
No

79.6.b - Do you routinely record this?
Yes
No

79.7.a - Is this important to make sense of outcome data?
Yes
No

79.7.b - Do you routinely record this?
Yes
No

79.8.a - Is this important to make sense of outcome data?
Yes
No

79.8.b - Do you routinely record this?
Yes
No

79.9.a - Is this important to make sense of outcome data?
Yes
No

79.9.b - Do you routinely record this?
Yes
No

79.10.a - Is this important to make sense of outcome data?
Yes
No

79.10.b - Do you routinely record this?
Yes
No

79.11.a - Is this important to make sense of outcome data?
Yes
No

79.11.b - Do you routinely record this?
Yes
No

79.12.a - Is this important to make sense of outcome data?
Yes
No

79.12.b - Do you routinely record this?
Yes
No

79.13.a - Is this important to make sense of outcome data?
Yes
No

79.13.b - Do you routinely record this?
Yes
No

79.14.a - Is this important to make sense of outcome data?
Yes
No

79.14.b - Do you routinely record this?
Yes
No

79.15.a - Is this important to make sense of outcome data?
Yes
No

79.15.b - Do you routinely record this?
Yes
No

79.16.a - Is this important to make sense of outcome data?
Yes
No

79.16.b - Do you routinely record this?
Yes
No

79.17.a - Is this important to make sense of outcome data?
Yes
No

79.17.b - Do you routinely record this?
Yes
No

79.18.a - Is this important to make sense of outcome data?
Yes
No

79.18.b - Do you routinely record this?

Yes

No
