# Supplementary material for: Maximising the Impact of Speech and Language Therapy for Children With Speech Sound Disorder (The MISLToe‐SSD) Study: Developing a Core Outcome Set (COS) for Routine Data Collection From UK NHS Speech and Language Therapy Services
Source: Int J Lang Commun Disord. 2026 Jan 9;61(1):e70188. doi: 10.1111/1460-6984.70188 (PMC12784794; doi:10.1111/1460-6984.70188)
Supplement: Supplementary file 3 — Supporting Information: jlcd70188‐sup‐0003‐SuppMat3MinimumDataset.pdf [file JLCD-61-0-s006.pdf]

SUPPLEMENTARY MATERIAL 3

Minimum Dataset

Common Data Elements presented to Expert Panel in Round One with percentage of respondents who considered they are important to collect in order to interpret the outcome data.

Common Data Element	Important to make sense of outcome data (%)	Routinely recorded (%)
Date of birth	88	98
Age at data collection point	100	97
Sex assigned at birth	52	79
Socio-economic status	58	9
Duration of session (minutes)	91	73
Spacing of session (per week)	98	92
Length of episode of care (weeks)	97	94
Total time in intervention (hours.mins)	80	34
Homework given	92	83
Location of sessions	58	94
Agent of intervention	100	93
Goals of intervention	98	97
Number of episodes of care	88	74
Diagnostic label 1	97	82
Diagnostic label 2	91	67
Birth order	26	30
Number of siblings	29	50
Languages spoken at home	94	95
