# Supplementary material for: Maximising the Impact of Speech and Language Therapy for Children With Speech Sound Disorder (The MISLToe‐SSD) Study: Developing a Core Outcome Set (COS) for Routine Data Collection From UK NHS Speech and Language Therapy Services
Source: Int J Lang Commun Disord. 2026 Jan 9;61(1):e70188. doi: 10.1111/1460-6984.70188 (PMC12784794; doi:10.1111/1460-6984.70188)
Supplement: Supplementary file 4 — Supporting Information: jlcd70188‐sup‐0004‐SuppMat4Round2survey.pdf [file JLCD-61-0-s003.pdf]

MISLToe-SSD UK Delphi Round Two (copy)

Page 1: Welcome to Round 2 of the MISLToe-SSD Delphi process

Maximising the **I**mpact of **S**peech and **L**anguage **T**herapy for children with **S**peech **S**ound **D**isorder (The MISLToe-SSD Study)

Thank you again for agreeing to be a member of the MISLToe-SSD expert panel. The aim of our work together is to reach a consensus agreement on a core set of outcomes from speech and language therapy (SLT) intervention for children with speech sound disorders (SSD). In addition to the outcomes of intervention, we aim to gain consensus on the way outcomes are assessed and which evidence based interventions most effectively lead to those outcomes.

Welcome to the second round of the Delphi process. Thank you for your contribution so far. In round one you made a judgement about outcomes. The items that reached over 50% positive consensus are here again for you to consider. You also gave us information about assessment of outcomes which we are taking forward here too. There are some new items to consider that were suggested (but not necessarily endorsed) by members of the panel.

In this round we will ask you to:

- make a judgement about SSD outcomes using the 5 point rating scale
- make judgements about assessments for SSD that are potential outcome measures for SSD
- give a rationale for some of your choices

Following this round we will provide feedback and you will have opportunity to change your responses. We will bring all the data to the online meeting of the expert panel and agree the final consensus there.

This Delphi Round will take 15- 20 minutes. There are two sections to this round: outcomes and assessments.

You can save your contribution and return at any time. Please submit this survey by midnight on Friday 5th May.

Page 2: Outcomes

The following statements relate to the outcomes of interventions for SSD. The outcomes included here all achieved at least 50% rating of moderately important, very important or essential in Round 1. Please consider them in relation to your clinical work, teaching and research.

The outcome is the ultimate or long term goal of one or more episodes of intervention for a child with SSD. For each outcome we will ask how important you think it is as an outcome of intervention for SSD. We will also ask you whether you think it should be considered as a primary outcome of intervention. Primary outcomes are the ones of most importance for clients and their families and/or speech and language therapists/pathologists (SLT/Ps). Secondary outcomes evaluate other beneficial effects of the intervention or explain additional effects of the intervention.

Population: Unless stated otherwise, please assume that we are considering the wider population of children who have received intervention from SLT/Ps for any and all subtypes of SSD of unknown origin. At this stage we are considering all of these children, so if an outcome is very important for a few children with SSD please rate with the same importance as an outcome that is very important for most of the children.

Timescale: Please remember that these are the desired outcomes at the end of one or more episodes of care, not for individual sessions.

1. What is your name? ✱ *Required*

2. What is your email address? ✱ *Required*

Page 3: Rating the importance of SSD intervention outcomes

3. Please rate the importance of the following statements as an outcome of intervention for SSD on the 5 point scale. For the statements you have rated 1 or 5 please explain why. You can also leave comments against any other statement if you wish to.

	✱ *Required*					
	1. Not at all important	2. Slightly important	3. Moderately important	4. Very important	5. Essential	Please explain your rating *Optional*
Increased speech intelligibility	○	○	○	○	○	
Increased confidence when talking	○	○	○	○	○	
Improved quality of life	○	○	○	○	○	
Improved communicative activity and participation	○	○	○	○	○	
Increased Percentage Consonants Correct (PCC)	○	○	○	○	○	
Increase in Percentage Phonemes Correct (PPC)	○	○	○	○	○	
Increase in Percentage Vowels Correct (PVC)	○	○	○	○	○	
Increase in Percentage of Words Correct (PWC)	○	○	○	○	○	
Increase in Percentage of Intelligible Utterances (PIU)	○	○	○	○	○	
Decrease in Proportion of Errors (PoE)	○	○	○	○	○	
Decrease in phonological variability	○	○	○	○	○	

Increased accuracy of target	○	○	○	○	○	
Increase in production of target sounds	○	○	○	○	○	
Increase in phonological awareness	○	○	○	○	○	
Increased stimulability	○	○	○	○	○	
Increase in number of phonemes	○	○	○	○	○	
Generalisation across linguistic units	○	○	○	○	○	
Generalisation across word position	○	○	○	○	○	
Generalisation to a new context	○	○	○	○	○	
Generalisation of known sounds	○	○	○	○	○	
Generalisation of the intervention target	○	○	○	○	○	
Generalisation related to the target (e.g., generalisation to other phonemes within and across sound classes)	○	○	○	○	○	
Increase in percentage child utterance attempts that are fully intelligible from language sample	○	○	○	○	○	

Page 4: Assessments: How we measure the outcomes

In Round 1, 100% of you said you would use the DEAP if it was available to you. We are not asking anything further about the DEAP in this round

4. In Round 1 you told us about the other assessments that are used within your services. Those with a large number of mentions are listed here. For children referred to SLT services with suspected SSD, please tell us what you know about these assessments that could be used to measure outcomes.

	Please check the boxes below to indicate **Yes** ✱ *Required*							
	I am not familiar with this assessment	I am familiar with this assessment	I have used this assessment	I would use this assessment if it was available	This assessment is suitable for baseline assessment	This assessment is suitable for outcome assessment	This assessment is suitable for progress assessment	Do you have any information to ac assessment? *Optiona*
Preschool and Primary Inventory of Phonological Awareness (PIPA)	☐	☐	☐	☐	☐	☐	☐	
The CLEAR Phonology screening Assessment	☐	☐	☐	☐	☐	☐	☐	
The Nuffield Dyspraxia Assessment	☐	☐	☐	☐	☐	☐	☐	
The South Tyneside Assessment of Phonology (STAP)	☐	☐	☐	☐	☐	☐	☐	
Intelligibility in Context Scale	☐	☐	☐	☐	☐	☐	☐	
The Newcastle Assessment of Phonological Awareness (NAPA or APAD)	☐	☐	☐	☐	☐	☐	☐	
The Clinical Assessment of Vowels-English System (CAV-ES)	☐	☐	☐	☐	☐	☐	☐	

Page 5: Final page

Maximising the Impact of Speech and Language Therapy for children with Speech Sound Disorder (The MISLToe-SSD Study)

Thank you for completing Round 2 of the MISLToe-SSD Delphi. We will be in touch soon with the results.
