# Supplementary material for: Maximising the Impact of Speech and Language Therapy for Children With Speech Sound Disorder (The MISLToe‐SSD) Study: Developing a Core Outcome Set (COS) for Routine Data Collection From UK NHS Speech and Language Therapy Services
Source: Int J Lang Commun Disord. 2026 Jan 9;61(1):e70188. doi: 10.1111/1460-6984.70188 (PMC12784794; doi:10.1111/1460-6984.70188)
Supplement: Supplementary file 5 — Supporting Information: jlcd70188‐sup‐0005‐SuppMat5panel_report.pdf [file JLCD-61-0-s005.pdf]

Maximising the Impact of Speech and Language Therapy for children with Speech Sound Disorder (The MISLToe-SSD Study)

Workstream 3: Reaching consensus through a modified Delphi process

The aim of the MISLToe-SSD Project is to develop a s Core Outcome Set (COS) for children with SSD. We are considering the measurable outcomes of speech and language therapy interventions for children with SSD.

Workstream 3 follows two previous workstreams that comprised an umbrella review of peer reviewed literature, two participatory workshops, a survey and card sorting activity. Figure 1 gives an overview of the modified Delphi process for the UK and International Expert Panels. The data presented here are from the UK Expert Panel only.

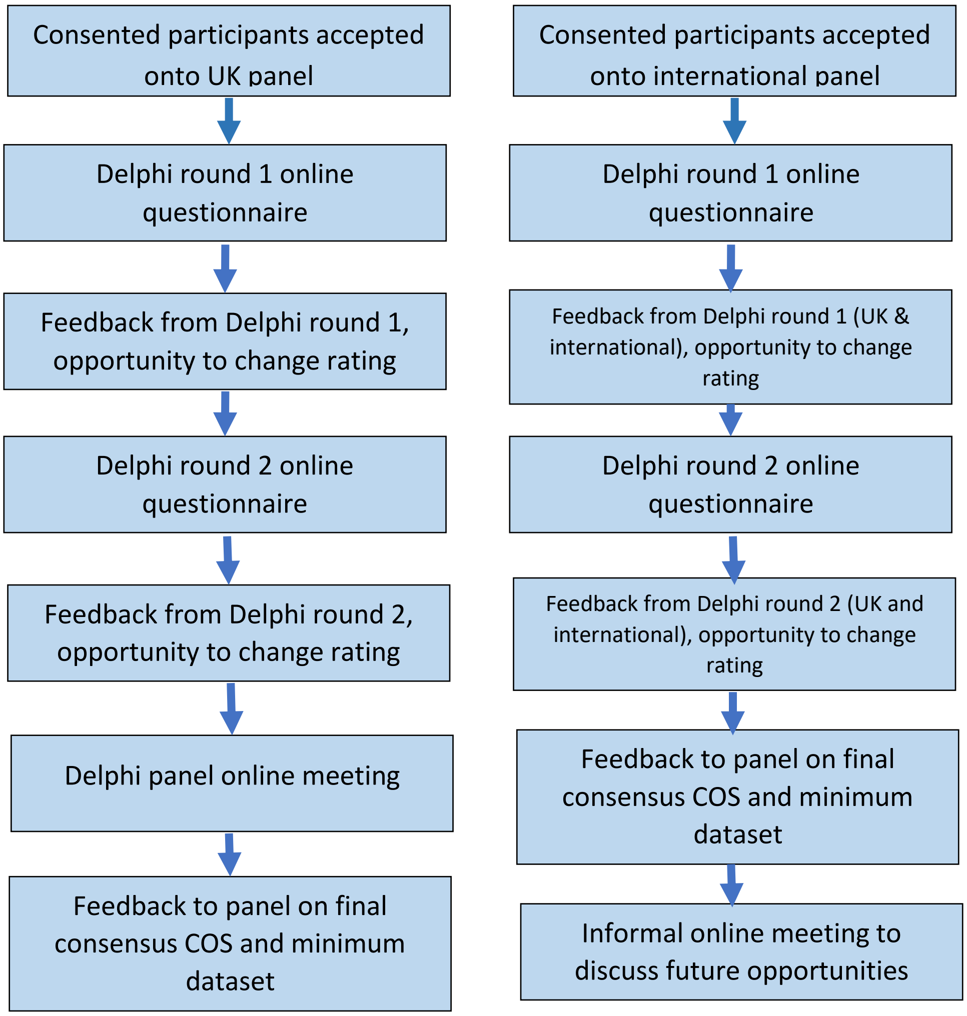

Figure 1. Flowchart for process of UK and international modified Delphi process

The UK Expert Panel

Seventy-one SLTs consented to take part in the modified Delphi process. They all confirmed that they were considered by others in their workplace as expert or specialist in SSD. Of these, five did not respond to the first round of the UK Delphi; their data is excluded from the demographic data

below. Sixty-six panel members took part in UK Delphi round 1, with 16 taking the opportunity to amend their initial responses in the feedback survey. Sixty-two UK panel members responded to the Round Two Delphi survey, with nine taking the opportunity to amend their initial responses in the feedback survey.

All four of the devolved UK countries (England, Scotland, Wales, Northern Ireland) were represented. The type of work undertaken by the in relation to children with SSD is set out in table 1.

Table 1. Type of work with children with SSD for Delphi Round 1 participants.

Type of work with children with SSD	Number of participants in Delphi Round 1 (n=66) (%)
Provide assessment and intervention for children with SSD	63 (95%)
Lead a clinical service for children with SSD	26 (39%)
Teach pre-registration (undergraduate or postgraduate) students about SSD	15 (23%)
Conduct research in SSD assessment or intervention	13 (20%)

Ten panel members (15%) had been qualified as an SLT for between four and nine years; 26 (39%) had been qualified for 10-19 years; 16 (24%) had been qualified for 20-29 years and 14 (21%) had been qualified for over 30 years. Twelve panel members (18%) had worked with children with SSD for between four and nine years; 29 (44%) had worked with children with SSD for 10-19 years; 14 (21%) had worked with children with SSD for 20-29 years and 11 (17%) had worked with children with SSD for over 30 years. The majority of the UK panel members were employed in the National Health Service (NHS), however, some had more than one employer, see Table 2.

Table 2. Employing organisations

Employer	Number of participants in Delphi Round 1 (n=66) (%)
NHS	53 (80%)
Independent/private practice	8 (12%)
Local Authority/Government	2 (3%)
Higher Education Institution (HEI) or University/College	9 (14%)
Other	1 (2%)

The Delphi surveys

A total of four surveys were sent out. In each of the two rounds there was a main survey then a follow up survey. The follow up survey was sent out with a summary of results from the main survey and an individual report for each participant. Panel members were invited to change their mind in the context of the other responses and to amend their responses to the survey.

Participants were reminded of the population and timescale for the outcomes as follows:

Population: Unless stated otherwise, please assume that we are considering the wider population of children who have received intervention from SLT/Ps for any and all subtypes of SSD of unknown

origin. At this stage we are considering all of these children, so if an outcome is very important for a few children with SSD please rate with the same importance as an outcome that is very important for most of the children.

Timescale: Please remember that these are the desired outcomes at the end of one or more episodes of care, not for individual sessions.

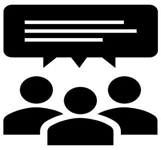

Discussion points that will be picked up in the Delphi Expert Panel meeting are highlighted in boxes like this one. **If you are unable to attend the meeting** and wish to contribute to the discussion you can do so by contributing to the Padlet (**click here.**)

Rating the importance of SSD intervention outcomes

The threshold for progression to the Round Two Delphi was 50% consensus at moderately important, very important or essential. Twenty-three outcome statements progressed to Round Two.

The threshold for progression of outcomes to the expert panel was 75% of responses at moderately important, very important or Essential.

Nineteen outcomes reached the consensus threshold in the UK Delphi Round Two. Table 3 shows both Round One and Round Two data. There was a high level of consensus within and across the two UK Delphi Rounds..

Table 3. Rating of intervention outcome statements as moderately important, very important or essential

SSD Intervention Outcomes	Round 1. 50% consensus threshold	Round 2. 75% consensus threshold
1. Increased speech intelligibility	100%	100%
2. Increased confidence when talking	91%	94%
3. Improved quality of life	89%	89%
4. Improved communicative activity and participation	98%	97%
5. Improved language	41%	n/a
6. Improved vocabulary	35%	n/a
7. Increased Percentage Consonants Correct (PCC)	89%	94%
8. Increase in Percentage phonemes correct (PPC)	80%	85%
9. Increase in Percentage vowels correct (PVC)	86%	87%
10. Increase in percentage of words correct (PWC)	73%	74%

SSD Intervention Outcomes	Round 1. 50% consensus threshold	Round 2. 75% consensus threshold
11. Increase in Percentage of intelligible utterances (PIU)	79%	84%
12. Decrease in proportion of errors (POE)	67%	65%
13. Decrease in phonological variability	86%	84%
14. Increased accuracy of target	88%	85%
15. Increase in production of target sounds	89%	82%
16. Increase in phonological awareness	86%	84%
17. Increased stimulability	94%	89%
18. Improved oromotor skills	24%	n/a
19. Increase in number of phonemes	85%	89%
20. Increase in egressive output	45%	n/a
21. Generalisation across linguistic units	85%	84%
22. Generalisation across word position	85%	87%
23. Generalisation to a new context	82%	87%
24. Generalisation of known sounds	73%	74%
25. Generalisation of the intervention target	91%	89%
26. Generalisation related to the target (e.g., generalisation to other phonemes within and across sound classes)	85%	85%
27. Increased mean length of utterance (MLU)	26%	n/a
28. Increase in percentage child utterance attempts that are fully intelligible from language sample	67%	71%
29. Parent report on increased structural complexity	41%	n/a
30. Parent report on increased phrase complexity	38%	n/a

85% Consensus or above 75%-84% consensus

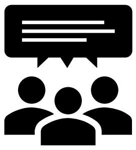

The outcomes marked in green in Table 3 are those we propose to take forward into the COS. Some will only be applicable to some of the SSD caseload, whilst others can be universally applied.

Two areas of outcome may prove to be more difficult to measure then the rest. We seek your opinion on how to label and measure these. They are:

- Items 2, 3 & 4, which relate to quality of life (QoL)
- Items 21, 22, 23, 25 &26, which all relate to generalisation

Primary and Secondary Outcomes

Delphi Round One Participants were asked to make a judgement as to whether the outcomes might be a primary outcome for children and families and/or for speech and language therapists, see table 4. These are outcomes that directly result from the speech and language therapy intervention that has been implemented during the episode of care. The primary outcome is the most important outcome of the intervention for clients and their families and/or speech and language therapists. Secondary outcomes evaluate other beneficial effects of the intervention or explain additional effects of the intervention.

Table 4. Round One Primary and secondary outcomes judgement

SSD Intervention Outcomes	Children/ Families Primary Outcome	SLT Primary outcome	Neither (i.e. secondary or other outcome)
1. Increased speech intelligibility	64 (97%)	59 (89%)	0
2. Increased confidence when talking	58 (88%)	41 (62%)	8 (12%)
3. Improved quality of life	52 (79%)	35 (53%)	13 (20%)
4. Improved communicative activity and participation	58 (88%)	47 (71%)	5 (8%)
5. Improved language	6 (9%)	3 (5%)	58 (88%)
6. Improved vocabulary	3 (5%)	3 (5%)	60 (91%)
7. Increased Percentage Consonants Correct (PCC)	2 (3%)	49 (74%)	16 (24%)
8. Increase in Percentage phonemes correct (PPC)	3 (5%)	47 (71%)	16 (24%)
9. Increase in Percentage vowels correct (PVC)	3 (5%)	43 (65%)	22 (33%)
10. Increase in percentage of words correct (PWC)	8 (12%)	37 (56%)	27 (41%)
11. Increase in Percentage of intelligible utterances (PIU)	15 (23%)	41 (62%)	21 (32%)

SSD Intervention Outcomes	Children/ Families Primary Outcome	SLT Primary outcome	Neither (i.e. secondary or other outcome)
12. Decrease in proportion of errors (POE)	8 (12%)	38 (58%)	27 (41%)
13. Decrease in phonological variability	1 (2%)	43 (65%)	23 (35%)
14. Increased accuracy of target	26 (39%)	52 (79%)	11 (17%)
15. Increase in production of target sounds	29 (44%)	53 (80%)	11 (17%)
16. Increase in phonological awareness	6 (9%)	43 (65%)	21 (32%)
17. Increased stimulability	12 (18%)	51 (77%)	15 (23%)
18. Improved oromotor skills	2 (3%)	8 (12%)	53 (80%)
19. Increase in number of phonemes	15 (23%)	45 (68%)	20 (30%)
20. Increase in egressive output	2 (3%)	19 (29%)	47 (71%)
21. Generalisation across linguistic units	3 (5%)	27 (41%)	37 (56%)
22. Generalisation across word position	6 (9%)	32 (48%)	31 (47%)
23. Generalisation to a new context	18 (27%)	36 (55%)	27 (41%)
24. Generalisation of known sounds	15 (23%)	30 (45%)	34 (53%)
25. Generalisation of the intervention target	28 (42%)	49 (74%)	14 (21%)
26. Generalisation related to the target (e.g., generalisation to other phonemes within and across sound classes)	5 (8%)	35 (53%)	29 (44%)
27. Increased mean length of utterance (MLU)	5 (8%)	1 (2%)	60 (91%)
28. Increase in percentage child utterance attempts that are fully intelligible from language sample	19 (29%)	31 (47%)	29 (44%)
29. Parent report on increased structural complexity	8 (12%)	5 (8%)	54 (82%)
30. Parent report on increased phrase complexity	9 (14%)	5 (8%)	54 (82%)

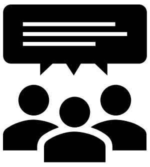

How do we arrive at one (or at most a few) primary outcome?

Considering the scores above, are there any outcomes that stand out as a primary outcome?

Do we only take forward those which can be universally applied?

Do we allocate these as Primary outcomes and the others as Secondary?

Outcome measures (assessments)

A diagnostic protocol was developed in Workstream 2. We will tell you more about this in the meeting and you can read about it here. In addition to assessment that provides an accurate, if evolving, diagnosis, it is necessary to measure the outcomes of interventions to know if our intervention has been successful in achieving its aims. This may overlap with some of the diagnostic protocol but may also include different assessments that measure specific outcomes of intervention.

Thirty-four assessments identified in the umbrella review were presented for the expert panel.

Fewer than 49% of the UK expert panel knew of the following assessments:

1. Arizona Articulation Proficiency Scale (AAPS) (0%)
2. Bankson-Bernthal Test of Phonology (BBTOP) (2%)
3. Children's Test of Nonword Repetition (Gathercole) (41%)
4. Comprehensive Test of Phonological Processing – second edition (CTOPP-2) (15%)
5. Comprehensive Test of Phonological Processing and Print Processing (0%)
6. Computer-Based Phonological Awareness Assessment (0%)
7. Computerized Articulation and Phonology Evaluation System (CAPES) (2%)
8. Denver Articulation Screening Exam (3%)
9. Goldman-Fristoe Test of Articulation (GFTA) (43%)
10. Glaspey Dynamic Assessment of Phonology (GDAP) (6%)
11. Grammar and Phonology Screening (GAPS) (8%)
12. Hodson Assessment of Phonological Patterns-3 (HAPP-3) (18%)
13. Individual Growth Development Indicator: Rhyming (2%)
14. McDonald Screening Deep Test of Articulation (0%)
15. Oral speech mechanism screen examination (OSMSE) (8%)
16. Phonological Abilities Test (Muter) (3%)
17. Phonological Assessment Battery (PhAB) (45%)
18. Phonological Assessment of Child Speech (PACS) (40%)
19. Phonological Awareness Literacy Screening– PreK (PALS-PreK) (0%)
20. Phonological Awareness Test (Robertson and Salter) (9%)
21. Phonological Knowledge Protocol (PKP) (0%)
22. Phonological Variability Test (0%)
23. Scaffolding Scale of Stimulability (SSS) (6%)
24. School Speech Questionnaire (Bergman) (2%)
25. Sutherland Phonological Awareness Test–Revised (6%)
26. Syllable Repetition Task (Shriberg et al.) (18%)

27. Test of Phonological Awareness–Second Edition: Plus Test of Preschool Early Literacy (TOPA-2+) (6%)
28. Templin-Darley Articulation Screening Test (0%)
29. Test of polysyllables (Gozzard et al.) (11%)
30. Verbal Motor Production Assessment for Children (Hayden and Square) (5%)
31. Word Complexity Measure (Stoel-Gammon) (5%)

The following assessments were known to over 50% of the expert panel:

1. Diagnostic Evaluation of Articulation and Phonology (DEAP) (100%)
2. Edinburgh Articulation Test (EAT) (50%)
3. Preschool and Primary Inventory of Phonological Awareness (PIPA) (60%)

Of these, the DEAP would be used by 100% of panel members if they had access to it; 98% considered it suitable as a baseline and outcome measure; 95% considered it suitable for measuring progress. The EAT was not considered suitable as a measure and only 6% said they would use it if they had access to it. The PIPA was considered as suitable as a baseline measure (52%) but only 49% would use it if available. Taking 50% use if available as the threshold, the DEAP, including the DEAP diagnostic Screen and the DEAP Toddler Phonology Test, goes forward as the favoured assessment at all stages for assessment of phonology and articulation.

A list of 37 assessments that were not identified by the umbrella review but were in use by panel members was generated and considered for inclusion in Round Two. Thirty-one of those assessments were mentioned by fewer than 4 people (20 mentioned only once). Those with 6 or more mentions, went forward to Round Two (see table 5).

Table 5. Additional assessments identified by panel members

Name of assessment	Number of mentions
CLEAR Phonology Screening Assessment	29
Nuffield Dyspraxia Assessment (NDA)	28
South Tyneside Assessment of Phonology (STAP)	23
Newcastle Assessment of Phonological Awareness (NAPA) (previously the APAD)	14
Intelligibility in Context Scale (ICS)	12
Clinical Assessment of Vowels-English System (CAV-ES)	6

Seven assessments were presented for consideration in Delphi Round 2. These included the six identified as additional assessments in Round One. The PIPA, identified as a baseline measure in Round One, was included to explore further the Panel's judgement on this assessment. Table 6 shows the percentage outcomes for the seven assessments. The clear distinction between knowledge and use was blurred in this survey due to the removal of the Round One directional pathway that only allowed panel members to make further responses if they were familiar with the assessment.

Table 6 Additional outcome measures

	PIPA	CLEAR	NDA	STAP	ICS	NAPA (formerly APAD)	CAV-ES
I have used this assessment	34%	71%	81%	73%	53%	35%	32%
I would use this assessment if it was available	6%	23%	44%	16%	37%	29%	24%
Suitable for baseline assessment	26%	55%	79%	50%	63%	44%	37%
Suitable for outcome assessment	13%	40%	73%	35%	61%	37%	34%
Suitable for progress assessment	15%	44%	73%	42%	53%	45%	37%

We propose to have:

- the DEAP as the only assessment for phonology and articulation.
- The Intelligibility in Context Scale as the only assessment for intelligibility
- the Nuffield Dyspraxia Assessment as an assessment for children suspected of having CAS (do we need a protocol for deciding this so that everyone uses the same criteria?)
- the NAPA as the assessment for phonological awareness

Language assessment

The majority (92%) of UK Expert Panel members routinely assess some aspect of language when children are referred primarily for SSD. Sixty-one percent routinely screen for language difficulties; 21% assess only if language delay or disorder Is flagged; 11% assess language in depth.

Should a language screen be a routine part of assessment of children presenting with SSD?

If so, what is a suitable tool? Should we also use it to collect a connected speech sample e.g. a sentence repetition task?

Minimum dataset

The Following common data elements were presented to the UK Expert Panel (table 4). Many of these are routinely collected or can easily be derived from routinely collected data in online data collection systems, we have highlighted those in blue. The two data elements that we do not need to collect are highlighted in orange.

Table 4 Common data elements

Common Data Element	Important to make sense of outcome data (%)	Routinely recorded (%)
Date of birth	88	98
Age at data collection point	100	97
Sex assigned at birth	52	79
Socio-economic status (postcode)	58	9
Duration of session (minutes)	91	73
Spacing of session (per week)	98	92
Length of episode of care (weeks)	97	94
Total time in intervention (hours.mins)	80	34
Homework given	92	83
Location of sessions	58	94
Agent of intervention	100	93
Goals of intervention	98	97
Number of episodes of care	88	74
Diagnostic label 1	97	82
Diagnostic label 2	91	67
Birth order	26	30
Number of siblings	29	50
Languages spoken at home	94	95

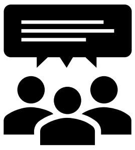

Each of these common data elements will be collected in an identical way for everyone using the COS e.g. date of birth will be in the same date format for everyone.

There are important issues around recording diagnosis. We will discuss these when we discuss the diagnostic protocol.
