# Supplementary material for: Maximising the Impact of Speech and Language Therapy for Children With Speech Sound Disorder (The MISLToe‐SSD) Study: Developing a Core Outcome Set (COS) for Routine Data Collection From UK NHS Speech and Language Therapy Services
Source: Int J Lang Commun Disord. 2026 Jan 9;61(1):e70188. doi: 10.1111/1460-6984.70188 (PMC12784794; doi:10.1111/1460-6984.70188)
Supplement: Supplementary file 6 — Supporting Information: jlcd70188‐sup‐0006‐SuppMat6Original_Protocol.pdf [file JLCD-61-0-s001.pdf]

Supplementary Material 6

Original protocol for Workstream 3 of the MISLToe_SSD study

Title: **M**aximising the **I**mpact of **S**peech and **L**anguage **T**herapy for children with **S**peech **S**ound **D**isorder (The MISLToe-SSD) Study (NIHR202766)

Workstream 3: Final consensus on COS and minimum dataset

Aim: To gain final consensus on items to be included in COS and minimum dataset, through a two-round modified Delphi exercise and expert panel meeting.

Participants: Purposively sampled expert panel drawn from academic, clinical specialist and our PPI group and RCSLT networks (n≤60).

Methods and analyses: *Round 1:* A comprehensive list of outcome measurement/analysis instruments generated in workstream 1 will be categorised and presented in an online survey for rating using a Likert scale. Participants will be invited to comment on their decision and offer revisions to wording. Analysis: Ratings will be analysed using descriptive statistics. Comments will be categorised and used to inform changes to items to take forward to next round. Feedback to participants will be given on their individual response in relation to the whole group and anonymised versions of all comments. *Round 2:* Outcome measurement/analysis instruments with an 'agree' rating from ≥50% of panel members will be presented for rating. Consensus will be defined a priori as a rating of 'agree' by ≥75% of participants. Participants will be invited to comment on their decision and offer revisions to wording. Analysis: Ratings will be analysed using descriptive statistics. Participants will be given feedback as in Round 1.

Panel meeting: The Delphi exercise will be followed by a virtual meeting of the panel using the outcome of round 2, to agree the final COS and minimum dataset. It is anticipated that 40 members of the panel will attend the meeting. Quality assessment of outcome measures will be conducted in alignment with the COnsensus-based Standards for the selection of health Measurement Instruments (COSMIN) protocol.

Deliverables: Finalised content for COS and minimum dataset.
